# The N-Terminal Domain of Bfa1 Coordinates Mitotic Exit Independent of GAP Activity in *Saccharomyces cerevisiae*

**DOI:** 10.3390/cells11142179

**Published:** 2022-07-12

**Authors:** Yan Li, Kiwon Song

**Affiliations:** Department of Biochemistry, College of Life Science and Biotechnology, Yonsei University, Seoul 03722, Korea; liyan900506@hotmail.com

**Keywords:** spindle misorientation, SPOC, Bfa1, microtubule hyper-elongation, spindle pole body, mitotic exit, budding yeast

## Abstract

The spindle position checkpoint (SPOC) of budding yeast delays mitotic exit in response to misaligned spindles to ensure cell survival and the maintenance of genomic stability. The GTPase-activating protein (GAP) complex Bfa1–Bub2, a key SPOC component, inhibits the GTPase Tem1 to induce mitotic arrest in response to DNA and spindle damage, as well as spindle misorientation. However, previous results strongly suggest that Bfa1 exerts a GAP-independent function in blocking mitotic exit in response to misaligned spindles. Thus, the molecular mechanism by which Bfa1 controls mitotic exit in response to misaligned spindles remains unclear. Here, we observed that overexpression of the N-terminal domain of Bfa1 (Bfa1-D16), which lacks GAP activity and cannot localize to the spindle pole body (SPB), induced cell cycle arrest along with hyper-elongation of astral microtubules (aMTs) as Bfa1 overexpression in Δ*bub2*. We found that Δ*bub2* cells overexpressing Bfa1 or Bfa1-D16 inhibited activation of Mob1, which is responsible for mitotic exit. In anaphase-arrested cells, Bfa1-D16 overexpression inhibited Tem1 binding to the SPB as well as Bfa1 overexpression. Additionally, endogenous levels of Bfa1-D16 showed minor SPOC activity that was not regulated by Kin4. These results suggested that Bfa1-D16 may block mitotic exit through inhibiting Tem1 activity outside of SPBs. Alternatively, Bfa1-D16 dispersed out of SPBs may block Tem1 binding to SPBs by physically interacting with Tem1 as previously reported. Moreover, we observed hyper-elongated aMTs in *tem1-3*, *cdc15-2,* and *dbf2-2* mutants that induce anaphase arrest and cannot undergo mitotic exit at restrictive temperatures, suggesting that aMT dynamics are closely related to the regulation of mitotic exit. Altogether, these observations suggest that Bfa1 can control the SPOC independent of its GAP activity and SPB localization.

## 1. Introduction

In the budding yeast *Saccharomyces cerevisiae*, the mitotic spindle must be correctly aligned along the mother bud polarity axis to ensure genomic stability. Chromosome segregation during mitosis is closely related to spindle position, which is guided by two important mechanisms: the Kar9 and dynein pathways. In the early phase of the cell cycle, Kar9 connects astral microtubules (aMTs) to actin cables at the cortex along with the MT-binding protein Bim1 (EB1 ortholog) and the type V myosin motor protein Myo2 to move the spindle to the bud neck [1,2,3]. A cortex-anchored motor dynein then continuously walks toward the minus end of aMTs and pulls aMTs to the bud cortex [4,5]. These pathways regulate the binding of the cortex and aMTs to guide the bud-directed spindle pole body (SPB: functional equivalent of the centrosome) through the bud neck and correctly orient the spindle. Mitotic exit occurs after chromosomes are properly segregated between the mother and the bud.

In late anaphase, components of the mitotic exit network (MEN) become active at the SPBs and release a key phosphatase, Cdc14, from the nucleolus to trigger mitotic exit in cells with properly aligned spindles [6,7]. The GTPase Tem1, a major component of the MEN, asymmetrically localizes to the bud-directed daughter SPB (dSPB) from early anaphase, which contributes to the recruitment of the Hippo-like kinase Cdc15 at the dSPB [8,9]. Active Cdc15 then localizes at both SPBs and phosphorylates the scaffold protein Nud1 to create a phospho-docking site for the large tumor suppressor (LATS)-related kinase Dbf2 and its coactivator Mob1 on the scaffold protein Nud1 [10,11]. Consequently, active Dbf2–Mob1 promotes the release of Cdc14 from the nucleolus [6,7], which dephosphorylates mitotic cyclin-dependent kinases (CDKs) and their substrates to induce mitotic exit [12,13]. However, some studies suggest that MEN activation is insufficient to trigger mitotic exit. Fusion of Tem1 or Cdc15 to SPB induces MEN activation in the early stage but does not trigger premature mitotic exit [14]. Additionally, the timing of mitotic exit in cells overexpressing Cdc15 is the same as that in wild-type cells, although Cdc15 overexpression can promote mitotic exit in Tem1-defective cells in response to spindle misorientation [14,15].

A surveillance mechanism called the spindle position checkpoint (SPOC) delays mitotic exit in response to spindle misorientation to ensure cell survival and maintenance of genomic stability. In cells with misaligned spindles, the cortical kinase Kin4 in the mother cell localizes to both SPBs within the mother cell cytoplasm to activate the GTPase-activating protein (GAP) Bfa1–Bub2 complex [16,17,18]. As a result, the two-component GAP complex Bfa1–Bub2 inhibits GTPase Tem1 and MEN activities to induce mitotic arrest [19]. However, previous results showed that Bfa1 plays a GAP-independent role in blocking mitotic exit in response to misaligned spindles [20,21]. Ro et al. [21] reported that Bfa1 overexpression arrests the cell cycle in Δ*bub2* cells and that the interaction between Tem1 and Cdc15 increased in the absence of Bfa1. Additionally, Kim et al. [22] showed that the overexpressed N-terminal domain of Bfa1, which cannot bind to Bub2, induces anaphase arrest similar to wild-type Bfa1. Moreover, the GAP-defective mutant *BFA1^W422A^* showed SPOC activity in Δ*bim1* cells with misoriented spindles [20]. Thus, the molecular mechanism by which Bfa1 controls mitotic exit in response to misaligned spindles remains unclear.

Bfa1–Bub2 GAP activity and MEN activation are mainly regulated by the Polo-like kinase Cdc5, the guanine nucleotide exchange factor (GEF) Lte1, and the Kin4 kinase [17,18]. In cells with an accurate spindle position, the GEF Lte1 localized in the bud cortex directly activates the GTPase Tem1 and prevents entry of Kin4 to the bud compartment [23]. Cdc5 asymmetrically localizes at the dSPB of cells with correctly aligned spindles and phosphorylates Bfa1 to inhibit Bfa1–Bub2 GAP activity at the dSPB, thereby causing mitotic exit [24]. In the case of spindle misorientation, Kin4 phosphorylates Bfa1 at two serine sites (S150 and S180), thereby enhancing the exclusion of Bfa1–Bub2 from the SPBs to prevent mitotic exit [16,17,18,25,26]. However, the precise mechanism by which the misaligned spindle is sensed and transmitted to Kin4 to activate the SPOC remains uncertain.

In this study, we investigated whether and how Bfa1 regulates mitotic exit in the SPOC independent of its GAP activity and SPB localization in budding yeast.

## 2. Materials and Methods

### 2.1. Yeast Strains and Culture

The *S. cerevisiae* strains used in the present study are listed in Table 1. Genes were tagged or deleted using PCR-based homologous recombination [27,28,29]. *TUB1-GFP-URA3* (pAFs125) was digested with the *Stu* I restriction enzyme (Takara Bio Inc., Shiga, Japan), *pRS304-BFA1-3HA* or *pRS304-BFA1-D16-3HA* was digested with *EcoR* V (Takara Bio Inc.), and *pRS306-mCherry-TUB1* was digested with *Apa* I (Takara Bio Inc.), followed by integration into the chromosomal DNA of the designated yeast strains.

Cells were incubated in YPAD medium (1% yeast extract, 2% bacto-peptone, 100 mg/mL adenine, and 2% dextrose) or in synthetic complete (SC) drop-out medium containing yeast nitrogen base and the necessary supplements [22]. Overexpression of genes was induced by the *GAL10-1* promoter with 2% galactose, as described by Kim et al. [22].

### 2.2. Plasmid Construct

The plasmids used in this study are listed in Table 2. DNA cloning was performed as previously described [30]. *BUB2*, the *BFA1* domains, *KIN4*, *SNU13*, 3HA-tagged *BFA1* containing the *BFA1* promoter, and 3HA-tagged *BFA1-D16* containing the *BFA1* promoter were amplified by PCR using chromosomal DNA of the w303 strain as a template and the primers described in Table 3. The amplified PCR products were subcloned into pMW(L)20*-pGAL*, pMW(U)20*-pGAL*, pMW(U)20*-pGAL-GFP*, or pRS304, respectively. The restriction sites (all enzymes from Takara Bio Inc.) used for DNA cloning are listed in Table 3. *PMW(L)20-pGAL-BFA1^2A^* and *PMW(L)20-pGAL-BFA1-D16^2A^* mutants were constructed by PCR-based site-directed mutagenesis using *PMW(L)20-pGAL-BFA1* and *PMW(L)20-pGAL-BFA1-D16* as PCR templates and the primers shown in Table 3. pMW20(U) *-pGAL*, pMW20(U)*-pGAL*-*GFP*, and pMW(L)20*-pGAL* were CEN-based vectors with the *GAL10-1* promoter.

### 2.3. Spotting Assay

Cells were incubated to mid-log phase and serially diluted 10-fold onto plates containing glucose or galactose. The assay was performed by incubating cells at 25 °C or 30 °C for 2 to 4 days after spotting the cells.

### 2.4. Bimolecular Fluorescence Complementation (BiFC) Analysis

The targeted genes were C-terminally tagged using PCR-based homologous recombination [29]. Cells co-expressing VN-tagged Bik1 and VC-tagged Kip2 or VN-tagged Bfa1 and VC-tagged Bim1 were visualized using a Zeiss LSM880 (Carl Zeiss, Jena, Germany) confocal microscope and ZEN software (Carl Zeiss) with a 100× oil objective.

### 2.5. Fluorescence Microscopy and Image Quantification

To observe nuclear positions and anaphase cells, cells were mounted in 4′,6-diamidino-2-phenylindole (DAPI)-containing medium (VECTASHIELD, Vector Laboratories, Burlingame, CA, USA) after fixation and sonication, and 2 μL of stained cells was loaded onto a glass slide. To monitor live cells with fluorescent signals, 2 μL of log-phase cells was loaded onto a glass slide, and images were taken immediately. The images (Figures 2F and 3A, Figure S4A–E) were visualized using a Zeiss LSM880 (Carl Zeiss) confocal microscope and ZEN software (Carl Zeiss) with a 100× oil objective. Other images were visualized using an Axioplan2 (Carl Zeiss) microscope with a 100× objective. Images were captured using an Axiocam CCD camera (Carl Zeiss) and AxioVision software (Carl Zeiss).

All image data were quantified using Fiji ImageJ (ImageJ 2.3.0/1.53q developed byWayne Rasband et al., National Institutes of Health, USA; download from https://imagej.net/ on 18 April 2022). For measurement of aMT and spindle length, a segmented line in Fiji ImageJ was used. For quantification of fluorescent signal intensity, the images for each experiment were captured by the same fluorescence exposure time. The background of images was corrected using the BaSiC plugin in Fiji ImageJ, and automatic regularization was applied to dark-field and flat-field correction using predefined shading profiles as described [31]. The intensity of Mob1-GFP and Tem1-RFP at the SPB was quantified by measuring the maximum intensity value in a segmented region containing a dot-like signal colocalized with the SPB. To measure the fluorescent signal dispersed out of SPBs, the mean intensity of a segmented area with a fixed size (2.37 μm^2^) per cell was detected.

### 2.6. Co-Immunoprecipitation (Co-IP) and Western Blot

Cells were grown to the log phase and lysed in lysis buffer using a bead beater (Biospec, Bartlesville, OK, USA). The lysis buffer contained 50 mM Tris-HCl (pH 7.5), 150 mM NaCl, 1% Nonidet P-40, 2 mM EDTA, 5 mM MgSO4, 50 mM NaF, 100 mM β-glycerophosphate, 1 mM Na_3_VO_4_, 1 mM dithiothreitol, 1 mM phenylmethylsulfonyl fluoride, and protease-inhibitor cocktail (Roche, Basel, Switzerland). To precipitate HA-tagged Bim1, total lysates (5 mg in lysis buffer) were incubated with an anti-HA antibody at 4 °C for 2 h, followed by incubation with protein A agarose beads (Invitrogen, Carlsbad, CA, USA) at 4 °C for 2 h. Lysates were then boiled with sodium dodecyl sulfate (SDS) sample buffer [60 mM Tris-HCl (pH 6.8), 10% SDS, 5% 2-mercaptoethanol, 25% glycerol, and 0.1% bromophenol blue]. To detect HA-tagged Bim1 and Myc-tagged Kar9 and Kip2, the anti-HA antibody (1:3000; Santa Cruz Biotechnology, CA, USA) and anti-MYC antibody (1:5000; Cell Signaling Technology, Danvers, MA, USA) were used for Western blot analyses.

### 2.7. Statistical Analysis

Statistical analysis was performed with the Student’s *t*-test using GraphPad Prism (GraphPad Software, Inc., La Jolla, CA, USA). * *p* < 0.05, ** *p* < 0.01, *** *p* < 0.001, and **** *p* < 0.0001 indicate statistical significance, and ns (*p* > 0.05) indicates non-significance.

## 3. Results

### 3.1. Overexpression of Bfa1 Induces Growth Arrest with Hyper-Elongated aMTs

**Figure 1:**
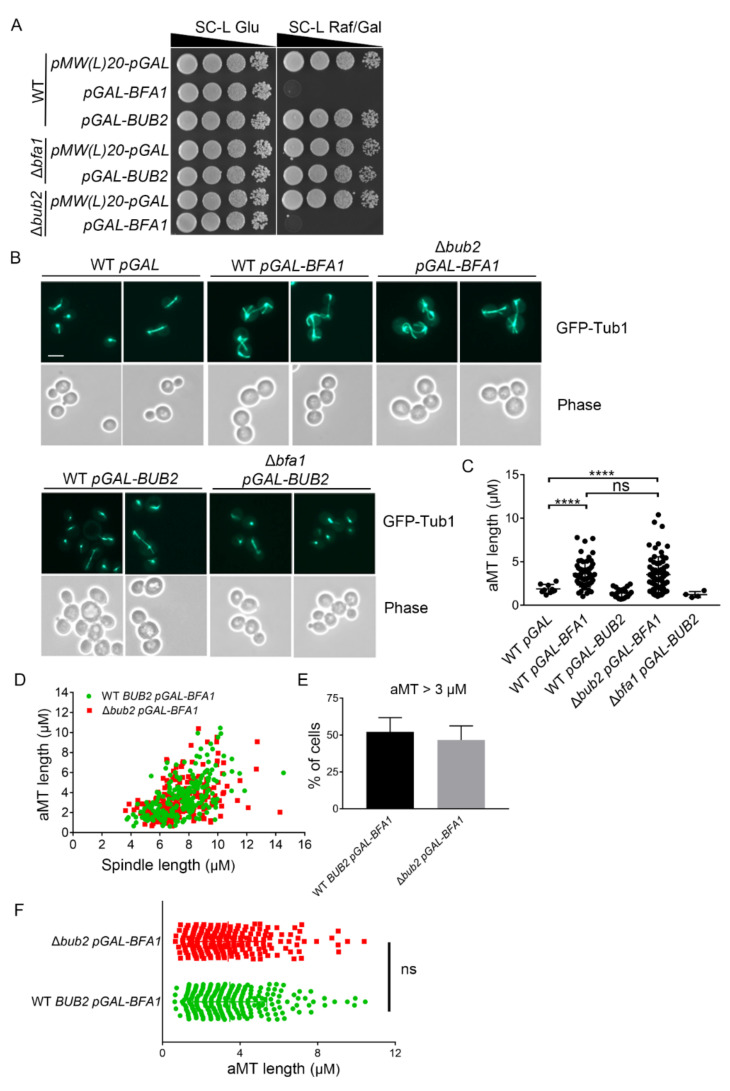
Bfa1 overexpression induces anaphase arrest along with hyper-elongated aMTs in Δ*bub2* as well as in wild-type cells. (**A**,**B**) Wild-type (YSK3278), Δ*bub2* (YSK3302), and Δ*bfa1* (YSK3279) strains whose chromosomes were integrated with *GFP-TUB1* were transformed with pMW(L)20-*pGAL-BFA1* and pMW(L)20-*pGAL-BUB2*, with pMW(L)20-*pGAL* used as vector control. (**A**) Cells were grown to mid-log phase, serially diluted 10-fold on both SC-L Glu (leucine-depleted SC medium with glucose) and SC-L Raf/Gal (leucine-depleted SC medium containing 2% raffinose and 2% galactose) plates, and further incubated at 30 °C. (**B**) Cells were incubated in SC-L Raf/Gal at 30 °C for 3 h to induce overexpression of Bfa1 or Bub2. GFP-Tub1 expression in these cells was observed, with images captured by an Axioplan2 fluorescence microscope with a 100× objective (Carl Zeiss). Scale bar, 5 μm. (**C**–**F**) The lengths of aMT and(or) the mitotic spindle were(was) detected in the indicated strain by Fiji ImageJ. The longest aMT per cell was measured. (**C**) The length of aMT per cell is presented as a single plot in the graph. The number of cells (*n* = 9, 57, 21, 69, and 4 for each indicated genotype) is shown in the graph. (**D**) The aMT and spindle length per cell are indicated as one dot in Bfa1-overexpressed WT and Δ*bub2*. (**E**) The percentage (%) of cells containing aMT length > 3 μm in (**D**) was counted. The data are presented as means ± standard deviation (SD) of three independent experiments. (**F**) The longest aMT per cell of (**D**) is presented. (**D**–**F**) A Total of 245 cells for WT and 217 cells for Δ*bub2* were analyzed by three independent experiments. (**C**,**F**) **** *p* < 0.0001 indicates statistically significant, and ns, not significant.

Previous studies reported that Bfa1 overexpression causes growth defects due to cell cycle arrest at anaphase and that this overexpression phenotype can be induced in cells depleted of *BUB2* [21,22]. First, we overexpressed *BFA1* or *BUB2* under the control of the *GAL10-1* promoter to confirm cell cycle arrest by spotting assays. Consistently, Bfa1 overexpression caused growth defects in Δ*bub2* cells, as well as in wild-type cells (Figure 1A). However, *BUB2* overexpression in Δ*bfa1* cells and wild-type cells did not result in any growth defects (Figure 1A).

Bub2 comprises GAP activity along with Bfa1 to inhibit MEN activity and induce mitotic arrest, but Bfa1 overexpression leads to growth defects independent of Bub2 as shown in Figure 1A. Thus, we examined the phenotype associated with Bfa1-overexpressing cells to identify a novel mechanism by which Bfa1 blocks mitotic exit. Monitoring of MTs via GFP-Tub1 revealed that Bfa1 overexpression induced hyper-elongated astral MTs (aMTs) and that this phenotype was not eliminated in Δ*bub2* cells (Figure 1B,C). We determined the length of mitotic spindles and the longest aMT per cell for each genotype. The length of mitotic spindles was considered around 3 μm for cells entering early anaphase and its length could be enriched to 8–10 μm at late anaphase [16,32]. We found that the aMTs in vector control or Bub2 overexpressed cells with anaphase spindles shorter than 3 μm (Figure 1C). However, about 50% of the wild-type (WT; 52.14 ± 9.66%) and Δ*bub2* (46.44 ± 9.30%) cells overexpressing Bfa1 showed that the length of aMTs was longer than 3 μm when the cells had anaphase spindles (Figure 1D,E). The results also showed that there was no significant difference in aMT length between the WT and Δ*bub2* cells overexpressing Bfa1 (Figure 1F). These results suggested that hyper-elongated aMTs induced by Bfa1 overexpression originated independently of Bfa1 GAP activity. Moreover, Bub2 overexpression neither suppressed mitotic exit nor induced the elongated aMT phenotypes observed in Bfa1-overexpressing cells (Figure 1A–C). These observations suggested that Bfa1 overexpression can induce cell cycle arrest and hyper-elongated aMT phenotypes independent of GAP.

### 3.2. Overexpression of the N-Terminal Domain of Bfa1 Induces Growth Arrest with Hyper-Elongated aMTs

**Figure 2:**
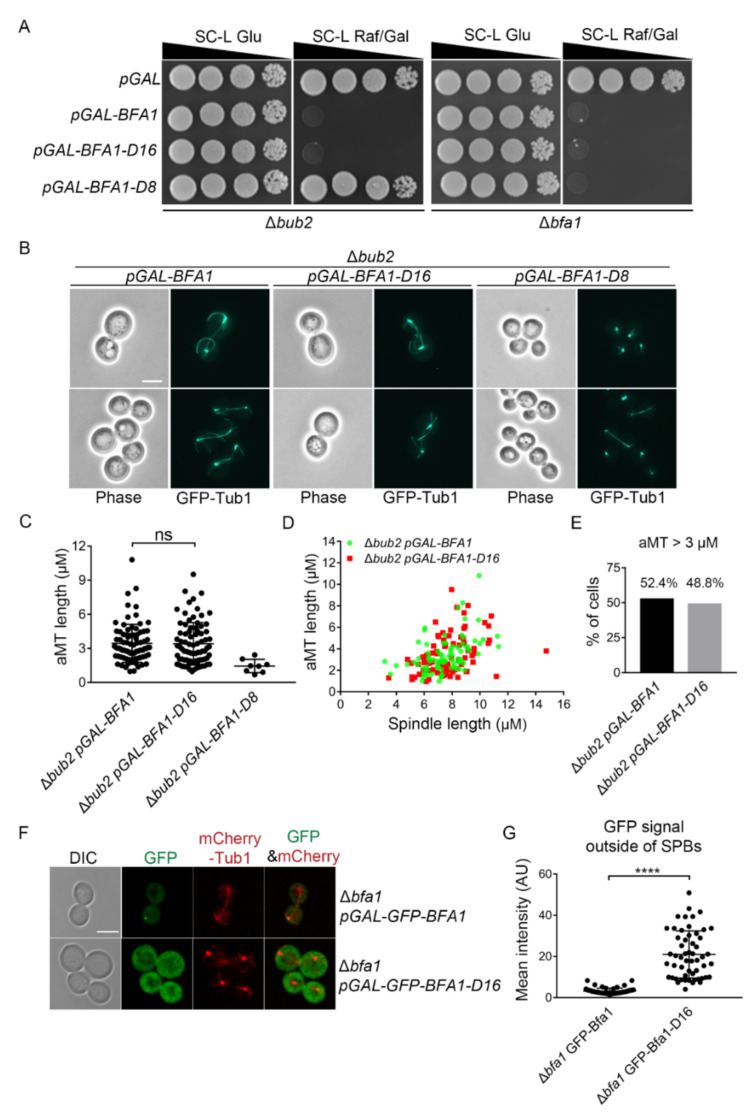
The N-terminal domain of Bfa1 (Bfa1-D16), which lacks SPB localization, induces anaphase arrest along with hyper-elongated aMTs in a *BUB2*-independent manner. (**A**,**B**) pMW(L)20-*pGAL-BFA1*, pMW(L)20-*pGAL-BFA1*-D16, and pMW(L)20-*pGAL-BFA1-D8* plasmids were used to transform the Δ*bub2* strain (YSK3302) and Δ*bfa1* strain (YSK3279), whose chromosomes were integrated with *GFP-TUB1*. (**A**) Serial dilutions of mid-log phase cells with the indicated plasmid were spotted on both SC-L Glu and SC-L Raf/Gal plates and incubated at 30 °C. The Δ*bub2* and Δ*bfa1* strains transformed with pMW(L)20-*pGAL* were used as vector control. (**B**) GFP-Tub1 was observed in Δ*bub2* cells transformed with the indicated plasmid following incubation at 30 °C for 3 h in SC-L Raf/Gal medium. Images were captured using an Axioplan2 fluorescence microscope with a 100× objective (Carl Zeiss). Scale bar, 5 μm. (**C**–**E**) The length of aMT and(or) the anaphase spindle in the indicated strain were(was) detected by Fiji ImageJ. The cells of Δ*bub2 pGAL-BFA1* (*n* = 82), Δ*bub2 pGAL-BFA1-D16* (*n* = 82), and Δ*bub2 pGAL-BFA1-D8* (*n* = 8) were determined. The longest aMT per cell was measured as aMT length. (**C**) Quantification of aMT length in the indicated strain. Each plot in the graph presents the length of the longest aMT per cell. (**D**) The aMT and spindle lengths per cell were indicated as a single plot in the graph. (**E**) The percentage (%) of cells carrying an aMT length > 3 μm in (**D**) was counted. (**F**) Localization of GFP-Bfa1 and GFP-Bfa1-D16 in Δ*bfa1* cells, respectively. Δ*bfa1* cells integrated with pRS304*-mCherry-Tub1* (YSK2874) and transformed with pMW(U)20-*pGAL*-*GFP-BFA1* or pMW(U)20-*pGAL*-*GFP-BFA1-D16* were grown to mid-log phase and incubated with SC-U medium (uracil-depleted SC) containing 2% galactose to induce the expression of Bfa1 or Bfa1-D16 at 25 °C for 3 h. Images were captured with an LSM 880 confocal microscope with a 100× objective (Carl Zeiss). Scale bar, 5 μm. (**G**) GFP signal was quantified by measuring the mean intensity of a segmented region (outside of SPBs) of a fixed area (2.37 μm^2^) per cell by Fiji ImageJ after background correction using the BaSiC plugin. A total number of 55 cells for each genotype are shown in the graph. (**C**,**G**) **** *p* <0.0001 indicates statistically significant, and ns, no significance.

We previously reported that overexpression of the N-terminal domain of Bfa1 (Bfa1-D16; 1–376 amino acids) induces anaphase arrest, even though it is defective in Bub2 binding and SPB localization (Figure S1A) [22]. To further evaluate the GAP-independent control of mitotic exit by Bfa1, we examined Bfa1-D16 in cells without Bub2. We observed that Bfa1-D16 expression under the control of the *GAL* promoter showed growth defects and hyper-elongated aMTs in Δ*bub2* cells, demonstrating a block of mitotic exit (Figure 2A–E). 48.8% of Δ*bub2* cells overexpressing Bfa1-D16 and having anaphase spindles carried aMTs longer than 3 μm, as observed in Δ*bub2* cells overexpressing Bfa1 (Figure 1E and Figure 2C–E). By contrast, overexpression of the C-terminal domain of Bfa1 (Bfa1-D8; 391–574 amino acids), which functions as a GAP with Bub2 and localizes at SPBs (Figure S1A) [19,22], did not induce growth arrest and hyper-elongated aMTs (aMT length > 3 μm in cells with anaphase spindles) in Δ*bub2* cells, even though it could induce growth arrest in the presence of Bub2 (Figure 2A–C). A previous report showed that neither wild-type Bfa1 without Bub2 nor Bfa1-D16 with Bub2 binds to SPBs [22,33]. Consistently, both Bfa1 and Bfa1-D16 were not detected at SPBs in Δ*bub2* cells, whereas Bfa1 localized to SPBs in the presence of Bub2 (Figure 2F and Figure S1B). In Δ*bfa1* cells, the intensity of GFP-Bfa1-D16 outside of SPBs was stronger than that of GFP-Bfa1 outside of SPBs (Figure 2F,G). Taken together, these results demonstrated that both Bfa1 and Bfa1-D16 in Δ*bub2* cells induced mitotic arrest with hyper-elongated aMTs, despite their cytoplasmic localization, and suggested that their mitotic arrest did not rely on their GAP activity or SPB localization.

Bfa1 binds to the SPB component Spc72 and is phosphorylated by Cdc5 and Kin4 [26]. In Δ*spc72* cells, SPB localization of Bfa1 was decreased [26]. To further confirm that Bfa1-D16 overexpression could induce mitotic arrest with cytoplasmic localization, we observed the localization of overexpressed Bfa1-D16 under the *GAL*-promoter in the Δ*spc72* strain. Overexpressed Bfa1-D16, which lacks SPB localization, continued to arrest cells with large buds and resulted in growth defects in Δ*spc72* cells (Figure S1C). These observations confirmed the ability of Bfa1-D16 dispersed out of SPBs to induce mitotic arrest.

### 3.3. Overexpressed Bfa1-D16 Inhibits MEN Activity Independent of GAP

**Figure 3:**
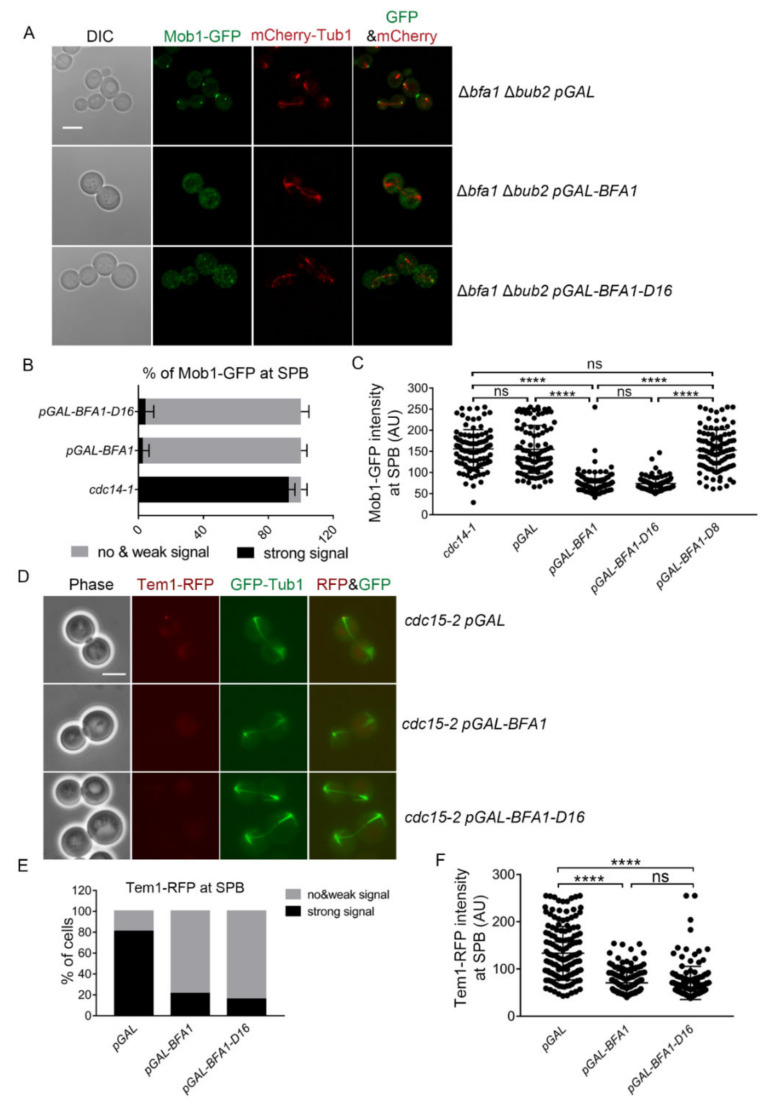
Bfa1-D16 overexpression prevents recruitment of MEN components to SPBs at late anaphase independent of its GAP activity. (**A**) Mob1-GFP localization was monitored with mCherry-Tub1 in Bfa1- or Bfa1-D16-overexpressing Δ*bfa1* Δ*bub2* cells. The Δ*bfa1* Δ*bub2* strain, whose chromosome was integrated with pRS304*-mCherry-Tub1* and contained *GFP*-tagged *MOB1* in its C-terminus (YSK3448), was transformed with pMW(L)20-*pGAL*, pMW(L)20-*pGAL-BFA1,* and pMW(L)20-*pGAL-BFA1-D16* plasmids. The cells were incubated with SC-L Raf/Gal at 25 °C for 3 h. Images were captured with an LSM 880 confocal microscope with a 100× objective (Carl Zeiss). Scale bar, 5 μm. (**B**,**C**) The Δ*bfa1* Δ*bub2* strain, with a chromosome containing *GFP*-tagged *MOB1* in its C-terminus (YSK3424), was transformed with pMW(L)20-*pGAL*, pMW(L)20-*pGAL-BFA1*, pMW(L)20-*pGAL-BFA1-D16*, and pMW(L)20-*pGAL-BFA1-D8* plasmids. The indicated cells were incubated with SC-L Raf/Gal at 25 °C for 3 h. (**B**) The percentage of cells with Mob1-GFP at SPBs in the *pGAL-BFA1*- and *pGAL-BFA1-D16*-expressing cells (*n* = 312, 412 for each genotype) was measured. The data were presented as means ± SD of three independent experiments. *cdc14-1* cells (*n* = 387) were used as a positive control of its localization to SPBs. *cdc14-1* (YSK1610) cells harboring *GFP*-tagged *MOB1* in its C-terminus were grown at 25 °C and then transferred to 37 °C for 3 h before counting the cells of Mob1 at the SPB. Images were captured using an Axioplan2 fluorescence microscope with a 100× objective (Carl Zeiss). (**C**) Mob1-GFP at SPB was quantified by measuring the maximum intensity in a segmented region of the dot-like signal (colocalized with SPB) after background correction using the BaSiC plugin in Fiji imageJ. A single plot of the graph presents one signal intensity per cell, and 100 cells for each genotype were analyzed. (**D**–**F**) Tem1-RFP signal was monitored with GFP-Tub1 in *cdc15-2* Δ*bfa1* cells expressing *pGAL-BFA1* or *pGAL-BFA1-D16*. The *cdc15-2* Δ*bfa1* strain, whose chromosome was integrated with *GFP-TUB1* and contained *RFP*-tagged *TEM1* in its C-terminus (YSK3451), was transformed with pMW(L)20-*pGAL*, *pGAL-BFA1*, and *pGAL-BFA1-D16*. Cells transformed with pMW(L)20-*pGAL* were used as controls of asymmetric localization of Tem1-RFP at SPBs during anaphase. (**D**) The images were captured using an Axioplan2 fluorescence microscope with a 100× objective (Carl Zeiss). Scale bar, 5 μm. (**E**) The percentage of cells with Tem1-RFP at the SPB in (**D**) is shown in the graph. Numbers of 177, 288, and 267 cells were counted for each genotype. (**F**) Tem1-RFP signal at SPB was quantified by measuring the maximum intensity in a segmented region where SPB was located after background correction of images by the BaSiC plugin in Fiji ImageJ. A total of 147 cells were counted for each genotype. (**C**,**F**) **** *p* < 0.0001 indicates a significant difference, and ns, no significant difference.

Recruiting Mob1 at the SPB is required for activation of the MEN pathway [10]. To determine whether Bfa1-D16 arrests mitotic exit via the known MEN pathway, we monitored Mob1-GFP localization in Bfa1-D16-overexpressed Δ*bub2* cells. Mob1-GFP localized to both SPBs of *cdc14-1* cells arrested in anaphase at a restrictive temperature when the upstream regulator of Mob1 in the MEN pathway was not inhibited (Figure S2A). In the absence of both *BFA1* and *BUB2* (Δ*bfa1*Δ*bub2* cells harboring vectors), strong Mob1-GFP signals were detected at both SPBs and the bud neck, and these cells were able to undergo mitotic exit and cytokinesis (Figure 3A,C and Figure S2A). Δ*bfa1* Δ*bub2* cells overexpressing the C-terminal Bfa1-D8 showed strong Mob1-GFP signals at both SPBs and the bud neck with no growth defects, given that these cells proceeded to mitotic exit and cytokinesis (Figure 2A, Figure 3C and Figure S2A). By contrast, overexpression of wild-type Bfa1 and Bfa1-D16 prevented the recruitment of Mob1-GFP to both SPBs in Δ*bfa1* Δ*bub2* cells, in which mitotic exit was blocked with growth defects (Figure 2A, Figure 3A–C and Figure S2A). Compared to *cdc14-1* cells, most Δ*bfa1* Δ*bub2* anaphase cells overexpressing either Bfa1 or Bfa1-D16 showed weak or no signals of Mob1-GFP at SPBs (Figure 3B). In addition, the Mob-GFP intensity at the SPBs in cells overexpressing Bfa1 or Bfa1-D16 was weaker than that in *cdc14-1* cells and in Δ*bfa1* Δ*bub2* cells overexpressing Bfa1-D8 or the vector control (Figure 3A–C and Figure S2A). Conversely, the Mob1-GFP intensity at a region outside of SPBs was stronger in Bfa1- or Bfa1-D16-overexpressing cells compared to that in *cdc14-1* cells (Figure S2B). These results strongly suggested that Bfa1-D16, devoid of GAP activity, controlled mitotic exit by blocking the activation of the MEN pathway in its downstream.

Then, how does Bfa1-D16 localized outside of SPBs inhibit Mob1 activity? Ro et al. [21] demonstrated that Bfa1 inhibits the interaction between Tem1 and Cdc15 in Δ*bub2* cells, whereas Bfa1 cannot localize to SPBs [33]. Bfa1-D16 physically interacts with Tem1, although it does not bind to Bub2 and SPBs [22]. Bfa1-D16 dispersed out of SPBs may also inhibit the upstream activity related to Mob1, such as binding Tem1 to sequester it outside of SPBs. Therefore, we monitored the localization of Tem1-RFP at anaphase in *cdc15-2* cells either overexpressing Bfa1 or Bfa1-D16 at a restrictive temperature. A strong Tem1-RFP signal was asymmetrically localized to SPBs in the *cdc15-2* anaphase cells harboring the vector only (Figure 3D–F). However, Tem1-RFP showed no or weak signals at SPBs of *cdc15-2* cells overexpressing Bfa1 or Bfa1-D16 (Figure 3D–F). These results demonstrated that the Bfa1-D16 blocks mitotic exit through inhibiting Tem1 localized outside of SPBs. It is possible that binding of Bfa1-D16 to Tem1 in the cytoplasm blocks Tem1 binding to SPBs, thereby inhibiting MEN activation.

Recently, de los Santos-Vela’zquez et al. [34] demonstrated that deletion of the small nucleolar ribonucleoprotein particle (snoRNP) assembly factor induces nucleolar hyper-condensation, leading to defects in Cdc14 release from the nucleolus and mitotic exit [34]. Additionally, they reported that Δ*bfa1* suppresses the synthetic lethality of Δ*lte1* with the deletion of *RSA1* (or *HIT1*), which is a box C/D snoRNP assembly factor [34]. Therefore, we questioned whether the full-length Bfa1 or Bfa1-D16 could block mitotic exit by inhibiting snoRNP independent of its GAP function. We examined whether the overexpression of Snu13, a component of box C/D snoRNP, could suppress the growth defects caused by Bfa1 overexpression in Δ*bub2* cells using spotting assays. The results showed that the growth defect caused by Bfa1 or Bfa1-D16 overexpression in Δ*bfa1* Δ*bub2* cells was not inhibited by Snu13 overexpression (Figure S2C,D). This result suggested that the mitotic arrest of Bfa1- or Bfa1-D16-overexpressing cells was not caused by inhibition of the snoRNP component.

### 3.4. Bfa1-D16 Exhibits SPOC Function in a Bub2-Independent Manner

**Figure 4:**
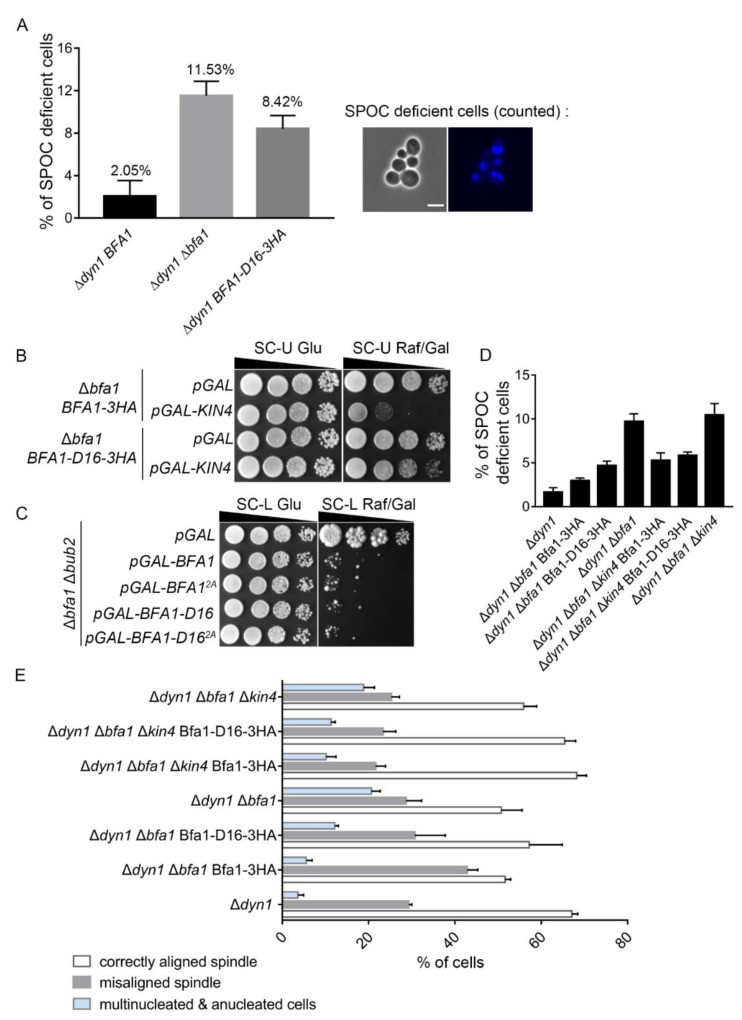
Bfa1-D16 exhibits SPOC functions in a *KIN4*-independent manner. (**A**) The SPOC activity of Bfa1-D16 was monitored in Δ*dyn1* cells. Δ*dyn1 bar1* cells expressing wild-type *BFA1* (YSK3356), *BFA1*-*D16* (YSK3421, the C-terminal domain of *BFA1* was replaced with 3HA epitope in the chromosomal *BFA1* locus of Δ*dyn1* cells), and Δ*dyn1* Δ*bfa1 bar1* cells (YSK1133) were used. Cells were grown to mid-log phase, synchronized by α-factor at 30 °C for 2–3 h, and transferred to YPDA medium for incubation at 16 °C for 18 h. The anucleated and multinucleated cells were counted after staining with DAPI. The percentage of SPOC-deficient cells was calculated as the number of anucleated and multinucleated cells relative to the total number of cells. The cells of each genotype were counted in four independent experiments (*n* = 1138, 1046, and 1096 for each indicated genotype), and the results are presented as the mean ± SD of four independent experiments. (**B**) Δ*bfa1* cells integrated with *BFA*1-*3HA* or *BFA1-D16-3HA* into the chromosome (YSK3422, YSK3423) were transformed with pMW(U)20-*pGAL* and pMW(U)20-*pGAL*-*KIN4*, respectively. Cells grown to mid-log phase were serially diluted (10-fold), spotted on SC-U Glu and SC-U Raf/Gal plates, and incubated at 25℃. (**C**) The Δ*bfa1* Δ*bub2* cells (YSK1879) transformed with pMW(L)20*-pGAL*, pMW(L)20-*pGAL-BFA1*, pMW(L)20*-pGAL-BFA1-D16*, pMW(L)20-*pGAL-BFA1^2A^*, and pMW(L)20*-pGAL-BFA1-D16^2A^* were serially diluted (10-fold), spotted on SC-L and SC-L Raf/Gal plates, and incubated at 25 °C. (**D**,**E**) The SPOC of Bfa1-D16 domain in *KIN4*-deleted cells. pRS304-*BFA1-3HA* or pRS304-*BFA1-D16-3HA* were integrated into the chromosome of Δ*dyn1* Δ*bfa1 bar1* (YSK1133) and Δ*dyn1* Δ*bfa1* Δ*kin4 bar1* (YSK2115) cells, respectively. The indicated cells (YSK3356, YSK3444, YSK3445, YSK1133, YSK3446, YSK3447, YSK2115) were synchronized by α-factor at 30 °C for 2–3 h. The cells were washed and released into YPAD medium, and further incubated at 16 °C for 18 h. The anucleated and multinucleated cells were counted after DAPI staining. (**D**) The percentage of SPOC-deficient cells was calculated as the number of anucleated and multinucleated cells relative to the total number of cells. The cells of each genotype were counted in three independent experiments (*n* = 1204, 846, 1124, 1089, 983, 914, and 1098 cells for each indicated genotype). (**E**) The percentage of cells with normally aligned spindle, misaligned, or anucleated/multinucleated cells in total anaphase cells (*n* = 552, 462, 436, 513, 513, 472, 608 for each genotype) of (**D**) was plotted. (**D**,**E**) The results were presented as the mean ± SD of three independent experiments.

We previously reported that the GAP activity of Bfa1 is required to block mitotic exit in response to DNA damage and spindle damage, but that the SPOC function of Bfa1 does not rely on GAP [20]. Thus, we investigated whether endogenously expressed Bfa1-D16 exerts SPOC activity in Δ*dyn1* cells with misoriented spindles. To express endogenous Bfa1-D16 under the *BFA1* promoter, we replaced the C-terminal domain of *BFA1* with 3HA epitopes in the chromosomal *BFA1* locus of Δ*dyn1* cells (YSK3421). Cells with the absence of the *DYN1* gene, which is a cytoplasmic dynein heavy chain required for spindle orientation, induce misaligned spindles [4,35]. The SPOC-deficient Δ*dyn1* cells undergo mitotic exit despite misaligned spindle sand become multinucleated or anucleated [9]. We counted the anucleated and multinucleated cells as SPOC-deficient cells in YSK3421 after DAPI staining and the percentage of SPOC-deficient cells calculated as the number of anucleated and multinucleated cells relative to the total number of cells. The proportion of SPOC-deficient cells among Bfa1-D16-expressing Δ*dyn1* cells was lower than that among Δ*dyn1* Δ*bfa1* double-knockout cells, even though Δ*dyn1* cells expressing only endogenous Bfa1-D16 showed more defects in SPOC function as compared with Δ*dyn1* cells expressing wild-type Bfa1 (Figure 4A). This result suggested that endogenous levels of Bfa1-D16 showed weak SPOC activity in Δ*dyn1* cells.

Kin4 activates Bfa1 by phosphorylating two serine sites (S150 and S180) in the N-terminal domain of Bfa1 (Bfa1-D16) in response to spindle misorientation [16,26]. To determine whether activation of the N-terminal domain of Bfa1 by Kin4 is necessary for the anaphase arrest induced by Bfa1 or Bfa1-D16 overexpression, we constructed strains endogenously expressing wild-type Bfa1 (YSK3422) and Bfa1-D16 (YSK3423) by integrating *BFA1* and *BFA1-D16* under the *BFA1* promoter in Δ*bfa1* cells and induced Kin4 overexpression in these strains. A previous report showed that Kin4 overexpression arrests cells expressing wild-type Bfa1 during anaphase [18]. Consistent with the former result, we found that Kin4 overexpression under the *GAL* promoter induced growth defects in cells endogenously expressing wild-type Bfa1 (Figure 4B). However, we found that cells overexpressing Kin4 grew well with expressing endogenous Bfa1-D16, although Bfa1-D16 contains the phosphorylation sites for Kin4 (Figure 4B). Moreover, we mutated the two phosphorylation target sites by Kin4 from serine to alanine (*S150A, S180A*) in wild-type *BFA1* (*BFA1^2A^*) and *BFA1-D16* (*BFA1-D16^2A^*). The previous result reported that Kin4 phosphorylation is blocked in the Bfa1^2A^ mutant and that Kin4 overexpression did not induce any growth toxicity in cells expressing Bfa1^2A^ [16]. We thus examined the mitotic arrest by overexpression of Bfa1^2A^ or Bfa1-D16^2A^ in Δ*bub2* cells and found that these cells still showed growth defects similar to those observed in Bfa1- or Bfa1-D16-overexpressing cells (Figure 4C). This result demonstrated that Bub2-independent mitotic arrest induced by Bfa1 or Bfa1-D16 is not regulated by Kin4.

To further examine whether Kin4 regulates Bfa1-D16 for mitotic arrest, we observed the SPOC of Bfa1-D16 in Δ*dyn1* Δ*kin4* cells. We constructed the strains whose chromosomal DNA of Δ*dyn1* Δ*kin4* Δ*bfa1* or Δ*dyn1* Δ*bfa1* cells were integrated with *BFA1-3HA* or *BFA1-D16-3HA* under the *BFA1* promoter. The percentage of SPOC-deficient cells was calculated as the number of anucleated and multinucleated cells relative to the total number of cells (Figure 4D) or the total number of anaphase cells (Figure 4E). Consistent with the result shown in Figure 4A, Bfa1-D16 conserved SPOC activity when compared to Δ*dyn1* Δ*bfa1*, although its SPOC activity was weaker than that of the cells expressing endogenous Bfa1 or Bfa1-3HA. SPOC activity was also conserved in Δ*dyn1* Δ*kin4* Δ*bfa1* cells expressing Bfa1-3HA under *BFA1* promoter, compared to Δ*dyn1* Δ*kin4* Δ*bfa1* cells (Figure 4D,E). Interestingly, Δ*dyn1* Δ*kin4* Δ*bfa1* cells expressing Bfa1-D16-3HA under *BFA1* promoter did not show as severe SPOC defects as Δ*dyn1* Δ*kin4* Δ*bfa1* cells, and the SPOC activity was similar to that of *KIN4* knockout cells with the wild-type Bfa1 (Figure 4D,E). Consistent with our observations, Δ*dyn1* Δ*kin4* cells did not lose the full SPOC activity in the previous report [36]. These results suggested that Kin4-independent SPOC activity of Bfa1 is conserved in Bfa1-D16, and supported that Bfa1-D16 is not regulated by Kin4.

### 3.5. Hyper-Elongated aMTs Are Conserved in the Cells of Late Mitotic Arrest

**Figure 5:**
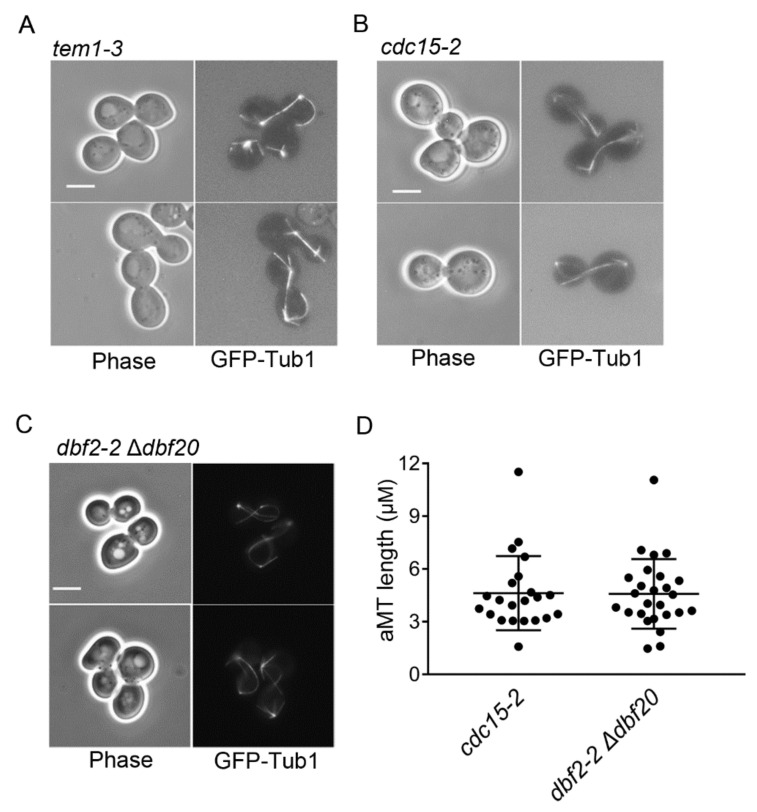
Hyper-elongated aMTs are present in *tem1-3*, *cdc15-2*, and *dbf2-2* Δ*dbf20* cells presenting a blocked mitotic exit. (**A**–**D**) The MT phenotypes in cells were visualized with GFP-Tub1. *GFP-TUB1* was integrated into the chromosomes of *tem1-3* (YSK556), *cdc15-2* (YSK553), and *dbf2-2 Δdbf20* (YSK3254) strains. Cells were grown to the mid-log phase at 25℃ and transferred to 37 °C for 3 h. Images were captured using an Axioplan2 fluorescence microscope with a 100× objective (Carl Zeiss). Scale bar, 5 μm. Cells of (**A**,**B**) were observed with YPAD medium and those of (**C**) were observed with SC medium. (**D**) The length of aMT in the indicated strain of (**B**) and (**C**) with anaphase spindles (spindle length > 3 μm) was detected by Fiji ImageJ, respectively. Cells of *cdc15-2* (*n* = 22), *dbf2-2* Δ*dbf20* (*n* = 26) were analyzed. The longest aMT per each cell was measured as aMT length. Each plot in the graph presents the length of the longest aMT per cell.

Because we consistently observed hyper-elongated aMTs in cells arrested in late mitosis by Bfa1 and Bfa1-D16 overexpression (Figure 1 and Figure 2), we questioned whether hyper-elongated aMTs are the cause or the result of late mitotic arrest. Thus, we investigated whether the hyper-elongated aMTs observed in Bfa1- and Bfa1-D16-overexpressing cells were the general phenotypes of cells in late mitotic arrest or specific to Bfa1-overexpressing cells. Specifically, we observed the aMT phenotypes associated with *tem1-3*, *cdc15-2*, and *dbf2-2* mutants at a restrictive temperature, where the MEN was inactivated, and cells were arrested at late mitosis. The result showed the presence of hyper-elongated aMT phenotypes (most cells showed an aMT length > 3 μm) in *cdc15-2* and *dbf2-2* mutants (Figure 5A–D).

To confirm that the hyper-elongated aMTs are not a cause of mitotic arrest, we determined whether the deletion of microtubule-associated proteins (MAPs) blocks mitotic arrest caused by Bfa1 overexpression or *cdc15-2* cells. The observation of GFP-Tub1 in Δ*kip2* and wild-type cells overexpressing Bfa1 revealed the disappearance of the hyper-elongated aMTs as a consequence of defective MT function due to the lack of *KIP2* but the arrest of the cells at anaphase (Figure S3A–C). Notably, the removal of Kip2 as well as other MAPs regulating MT dynamics did not rescue the growth arrest induced by Bfa1 overexpression (Figure S3C–E). These results suggested that late mitotic arrest caused by Bfa1 overexpression was not mediated by the hyper-elongated aMT phenotype. Furthermore, when we observed the aMTs in a *cdc15-2* Δ*kip2* double mutant at the restrictive temperature for *cdc15-2*, deletion of *KIP2* did not suppress the anaphase arrest induced by *cdc15-2* mutation (Figure S3F–H). These cells showed defects in aMT elongation but phenotypes similar to Bfa1 overexpression in Δ*kip2* cells. These results demonstrated that hyper-elongated aMTs represent conserved phenotypes of the block of mitotic exit and are the result of mitotic arrest induced by Bfa1 and Bfa1-D16 overexpression.

### 3.6. Overexpression of Hyper-Elongated aMTs by Bfa1 or Bfa1-D16 Is Not Mediated by Their Interaction with MAPs

Notably, we routinely observed hyper-elongated aMTs with anaphase spindles in cells overexpressing Bfa1 and Bfa1-D16 (Figure 1 and Figure 2), and aMTs were directly responsible for mitotic spindle orientation. In addition, *BFA1* has genetic interactions with several genes encoding MT-associated proteins (MAPs), such as *BIM1*, *BIK1*, *KAR9*, and *KAR3*, according to a synthetic genetic array [37]. Thus, to determine whether the hyper-elongated aMTs were directly associated with Bfa1- and Bfa1-D16, we used BiFC analysis to examine the interaction between Bfa1 and several MAPs. The BiFC assay is a reliable method for studying protein–protein interactions in yeast cells [29]. We first confirmed whether the BiFC assay could visualize the Kip2–Bik1 interaction (well-known interacting MAPs in yeast cells) (Figure S4A). We subsequently found that Bfa1 interacted with the MT-binding protein Bim1 according to the BiFC assay (Figure S4B). The Bfa1–Bim1 interaction was observed in one of the SPBs in WT cells but not visible in Δ*bub2* cells (Figure S4B–E).

Kar9 via its interaction with Bim1 mediates the capture of aMTs in the cortex [2,3]. Additionally, Scarfone et al. [36] reported that Bfa1 binding to SPBs via the Spc72–Bfa1 fusion protein affects the asymmetric distribution of Kar9. Because the hyper-elongated aMTs observed in Bfa1-overexpressing cells may be due to the disturbance of the connection between aMTs and the cell cortex, we evaluated whether Bfa1 overexpression inhibits the Kar9 interaction with Bim1 to affect aMT–cortex attachment. We detected the physical interaction between Kar9 and Bim1 in Bfa1-overexpressing cells by co-immunoprecipitation (Co-IP) assay and found that their interaction was not diminished in Bfa1-overexpressing cells compared to the vector control (Figure S4F). This result showed that Bfa1 overexpression likely did not block Kar9 binding to MTs.

Kip2 mediates MT stabilization by interacting with Bik1 (human CLIP-170 ortholog) and Bim1 [38]. Additionally, Chen et al. [39] reported that Δ*bfa1* affects Kip2 asymmetric loading onto bud-directed SPBs. Moreover, overexpression of Kip2 kinesin under the control of the *GAL* promoter resulted in hyper-elongated MTs [40], similar to that observed in Bfa1- and Bfa1-D16-overexpressing cells in the present study. Given the identified Bfa1–Bim1 interaction shown in Figure S4B, we examined whether Bfa1 overexpression enhances the binding of Kip2 and Bim1 to induce hyper-elongated MTs. Co-IP assay for Kip2 and Bim1 in Bfa1-overexpressing cells revealed that Kip2–Bim1 interaction was unaltered in Bfa1-overexpressing cells compared with that observed in the vector control (Figure S4G). These observations demonstrated that Bfa1 overexpression does not directly affect the Bim1–Kip2 and Bim1–Kar9 interactions to cause hyper-elongated aMTs, despite Bfa1 interaction with the MT-binding protein Bim1. Because full-length Bfa1 did not show functional connections with MAPs, we did not examine the same possibility for Bfa1-D16.

Given that a previous report showed that Bfa1 affects the asymmetric distribution of MAPs such as Kip2 and Kar9 [36,39] and based on the present results showing that Bfa1 interacts with Bim1, we determined whether Bfa1 regulates spindle position. We observed nuclear migration in Δ*bfa1* cells compared with that in wild-type cells. The cells synchronized with hydroxyurea were subsequently released into YPAD medium for 20 min and 60 min respectively, and their nuclear positioning was observed by DAPI staining to count the number of cells showing aberrant nuclear migration. We found that aberrant nuclear migration increased slightly (>3%) in Δ*bfa1* cells compared with that in the WT (Figure S5A,B), suggesting that *BFA1* plays a minor role in nuclear migration.

## 4. Discussion

In cases of spindle misorientation, the surveillance mechanism of budding yeast, SPOC, is activated to arrest the cell cycle until the spindle is properly oriented. This mechanism ensures the equal separation of genetic material into mother and daughter (bud) cells. The Bfa1–Bub2 GAP complex, a key component of SPOC, inhibits the GTPase Tem1 and MEN activities to induce mitotic arrest [19]. However, several previous studies suggested that Bfa1 can block mitotic exit in a GAP-independent manner [20,21]. Therefore, in the present study, we investigated the possible mechanisms by which Bfa1 regulates mitotic exit independent of GAP activity and SPB localization.

To investigate the GAP-independent function of Bfa1 in SPOC, we examined the N-terminal Bfa1-D16 domain. Overexpression of Bfa1-D16, which cannot bind to Bub2 and SPBs, induces anaphase arrest [22]. We showed that overexpressed Bfa1 or Bfa1-D16 in Δ*bub2* cells was not detectable on SPBs and arrested the cell cycle in late mitosis along with hyper-elongated aMTs (Figure 1 and Figure 2 and Figure S1B), whereas overexpression of Bub2 or the Bub2-dependent Bfa1-D8 domain did not induce cell cycle arrest (Figure 1 and Figure 2). These results suggested that Bfa1 or Bfa1-D16 can induce cell cycle arrest independent of Bub2 with their localization outside of SPBs. Furthermore, overexpression of either Bfa1 or Bfa1-D16-blocked Mob1 and Tem1 binding to SPBs in late mitotic cells (Figure 3). These results suggest that the GAP-independent Bfa1-D16 domain should inhibit MEN activity for arresting mitotic exit in the cytoplasm. We further evaluated the SPOC activity of endogenous Bfa1-D16 in Δ*dyn1* cells, finding minor SPOC activity by endogenous Bfa1-D16 in cells with misaligned spindles (Figure 4A,D,E). Then, how does Bfa1-D16 localized outside of SPBs inhibit MEN activity? Scarfone et al. [36] reported that Bfa1 binding to SPBs facilitates mitotic exit. Their study also showed that the Spc72–Bfa1 fusion protein recruits MEN components such as Tem1 and Cdc15, and that Δ*dyn1* cells expressing the fusion protein lack SPOC activity [36]. Additionally, Bfa1-D16 physically interacts with Tem1 by the yeast 2-hybrid, although it does not bind to Bub2 and SPB [22]. Therefore, Bfa1-D16 dispersed out of SPBs may inhibit the upstream activity such as Tem1 in the cytoplasm and (or) not facilitate binding of Tem1 or other MEN components to SPBs, thereby exerting SPOC activity.

The Kin4-phosphorylation sites of Bfa1 are distributed in Bfa1-D16, but we observed no growth arrest following Kin4 overexpression in cells expressing Bfa1-D16 (Figure 4B). Similarly, overexpression of the Kin4-phosphorylation-blocked mutants, Bfa1^2A^ and Bfa1-D16^2A^, induced growth arrest to the same degree as that observed in the overexpression of wild-type Bfa1 and Bfa1-D16 (Figure 4C). Furthermore, Bfa1-D16 conserved the SPOC activity in Δ*dyn1* Δ*bfa1* cells with the absence of Kin4 as well as in Δ*dyn1* Δ*bfa1* cells expressing Kin4 (Figure 4D,E). These data support that the SPOC activity of Bfa1-D16 is not regulated by Kin4. How does Bfa1-D16, which is not regulated by Kin4, conserve SPOC activity? The SPB component Spc72 is a platform for Bfa1 phosphorylation by Cdc5 and Kin4 [16,26]. Kin4 phosphorylates Bfa1 to block its inhibitory phosphorylation by Cdc5. Gryaznova et al. [26] demonstrated that Δ*spc72* cells retain SPOC activity, despite the inability of Kin4 to phosphorylate Bfa1; the inhibitory phosphorylation of Bfa1 by Cdc5 is diminished in Δ*spc72* cells. Our previous results showed that the GAP-defective mutant *BFA1^W422A^* exhibits SPOC activity in cells with misoriented spindles [20]. *BFA1^W422A^* lacks Cdc5 phosphorylation and delays mitotic exit during the unperturbed cell cycle [41]. Thus, the results of the present study and those of previous reports suggest that Bfa1-D16 might not be inhibited by Cdc5 and inhibit Tem1 outside of SPBs to conserve SPOC activity. It is possible that Bfa1 translocated from the SPB to the outside might delay the cell cycle by decreasing the MEN signal loading onto the SPB, or(and) by escaping inhibitory phosphorylation by Cdc5, or (and) by other unknown mechanisms.

Both *BFA1-D16* and *BFA1^W422A^* mutants are GAP-deficient and arrest the cell cycle following their overexpression, though their endogenous levels resulted in different degrees of SPOC activity. Bfa1^W422A^ showed a similar level of SPOC activity as wild-type Bfa1 [20], whereas Bfa1-D16 showed minor SPOC activity as shown in Figure 4. The difference between the two Bfa1 mutants is in their SPB localization. Bfa1^W422A^ localizes to SPBs and binds Bub2, even though the mutant lacks GAP activity [20]. In this study, we found that Bfa1 interacted with Bim1 in the SPB by using BiFC assay, but this signal was not observed in *BUB2*-depleted cells (Figure S4). Moreover, asymmetric localization of Bfa1 (or Bub2) to SPBs affects the asymmetric loading of MAPs, such as Kip2, to bud-directed SPBs [39]. Thus, it is likely that Bfa1 localized at SPBs binds not only to Nud1 but also Spc72, thereby increasing the efficient sensing of SPOC signals. These data suggest that the Bfa1 N-terminus (Bfa1-D16) exerts SPOC activity but may not sense spindle misalignment because of a defect in SPB binding.

In this study, we observed hyper-elongated aMTs in Bfa1- and Bfa-D16-overexpressing wild-type or Δ*bub2* cells arrested in late mitosis (Figure 1 and Figure 2). The deletion of several MAPs did not suppress anaphase arrest in Bfa1-overexpressing cells (Figure S3). These data suggest that the cell cycle arrest induced by Bfa1 overexpression is not mediated by hyper-elongated aMTs. Moreover, we found that the hyper-elongated MTs were conserved in MEN-inhibited cells, suggesting that the hyper-elongated aMTs are correlated with the blocking of mitotic exit (Figure 5). We subsequently determined whether Bfa1 overexpression directly affects MAPs to induce hyper-elongated aMTs. Because Bfa1 interacts with the MT-binding protein Bim1 by BiFC assay (Figure S4B), we investigated whether Bfa1 overexpression affects interactions between Bim1–Kip2 and Bim1–Kar9 to cause hyper-elongated aMTs by affecting MT–cortex attachment. However, Bfa1 overexpression did not affect the Bim1–Kip2 and Bim1–Kar9 interactions (Figure S4F,G). When MEN is inhibited in cells, the release of its downstream regulator, Cdc14, from the nucleus is blocked, thereby sustaining high CDK activity. Thus, MEN inactivation or active CDKs might contribute to the regulation of MAPs that affect MT dynamics and accompany hyper-elongated MTs [38,42,43,44].

## Figures and Tables

**Table 1:** Yeast strains used in this study.

Strains	Genotype	Source
W303	*MATa ade2-1 ura3-1 trp1-1 leu2-3,112 his3-11,15 can1-100*	S. Elledge
YSK3278	W303a *bar1 GFP-TUB1::URA3* (pAFS125)	This study
YSK3279	W303a *bar1* Δ*bfa1::KanMx6 GFP-TUB1::URA3* (pAFS125)	This study
YSK3302	W303a *bar1* Δ*bub2::KanMx6 -GFP-TUB1::URA3* (pAFS125)	This study
YSK556	W303a *tem1-3 GFP-TUB1::URA3* (pAFS125)	Laboratory stock
YSK553	W303a *cdc15-2 GFP-TUB1::URA3* (pAFs125)	Laboratory stock
YSK3322	*cdc15-2 GFP-TUB1::URA3* (pAFs125) Δ*kip2::KanMx6*	This study
YSK3254	*dbf2-2* Δ*dbf20::TRP1 GFP-TUB1::URA3 (pAFs125)*	This study
YSK3283	W303a *bar1 KIP2-VC::TRP1*	This study
YSK3284	W303a *bar1 KIP2-VC::TRP1 BIK1-VN::HIS3*	This study
YSK3150	W303a *bar1 BFA1-VN::HIS3*	This study
YSK3304	W303a *bar1 BIM1-VC::TRP1 BFA1-VN::HIS3*	This study
YSK3359	W303a *bar1 BIM1-VC::TRP1 BFA1-VN::HIS3 SPC42-CFP-KAN*	This study
YSK3360	W303a *bar1 BIM1-VC::TRP1 BFA1-VN::HIS3* pRS306*-mCherry-TUB1*	This study
YSK3361	W303a *bar1 BIM1-VC::TRP1 BFA1-VN::HIS3* Δ*bub2::KAN*	This study
YSK3268	W303a *bar1* Δ*bfa1::KanMx6 KAR9-9myc::hphNT1*	This study
YSK3269	W303a *bar1* Δ*bfa1::KanMx6 KAR9-9myc::hphNT1 BIM1-3HA::HIS5*	This study
YSK3243	W303a *bar1* Δ*bfa1::KanMx6 KIP2-9myc::hphNT1*	This study
YSK3244	W303a *bar1* Δ*bfa1:: KanMx6 KIP2-9myc-hphNT1 BIM1-3HA::HIS5*	This study
YSK3434	W303a *bar1 GFP-TUB1::URA3*(pAFs125) Δ*kip2:: KanMx6*	This study
YSK368	W303a *bar1*	A.Amon
YSK3344	W303a *bar1* Δ*kar9::KanMx6*	This study
YSK3345	W303a *bar1* Δ*bim1::KanMx6*	This study
YSK3346	W303a *bar1* Δ*kar3::KanMx6*	This study
YSK3347	W303a *bar1* Δ*cik1::KanMx6*	This study
YSK3348	W303a *bar1* Δ*kip2::KanMx6*	This study
YSK3349	W303a *bar1* Δ*bik1::KanMx6*	This study
YSK1610	*cdc14-1* Δ*bfa1::hphNT1 MOB1-GFP::KAN*	Laboratory stock
YSK3424	W303a Δ*bfa1::HIS3* Δ*bub2::URA3 MOB1-GFP::KAN*	This study
YSK3356	W303a *bar1* Δ*dyn1::LEU2*	This study
YSK3421	W303a *bar1* Δ*dyn1::LEU2 BFA1-D16-3HA::HIS3*	This study
YSK1133	W303a *bar1* Δ*bfa1::KanMx6* Δ*dyn1::LEU2*	Laboratory stock
YSK1879	W303a Δ*bfa1::HIS3* Δ*bub2::KAN*	Laboratory stock
YSK3270	W303a *bar1* Δ*bfa1::KanMx6*	This study
YSK3435	BY4741 Δ*spc72::KanMx6*	Laboratory stock
YSK3422	W303a *bar1* Δ*bfa1::KanMx6 pRS304-BFA1-3HA*	This study
YSK3423	W303a *bar1* Δ *bfa1::KanMx6 pRS304-BFA1-D16-3HA*	This study
YSK3444	W303a *bar1* Δ*bfa1::KanMx6* Δ*dyn1::LEU2* pRS304*-BFA1-3HA*	This study
YSK3445	W303a *bar1* Δ*bfa1::KanMx6* Δ*dyn1::LEU2* pRS304*-BFA1-D16-3HA*	This study
YSK2115	W303a *bar1* Δ*dyn1::LEU2* Δ*bfa1::KAN* Δ*kin4::hphNT1*	Laboratory stock
YSK3446	W303a *bar1* Δ*dyn1::LEU2* Δ*bfa1::KAN* Δ*kin4::hphNT1* pRS304-*BFA1-3HA*	This study
YSK3447	W303a *bar1* Δ*dyn1::LEU2* Δ*bfa1::KAN* Δ*kin4::hphNT1* pRS304-*BFA1-D16-3HA*	This study
YSK3451	W303a *cdc15-2* Δ*bfa1::HIS5 TEM1-RFP::KAN GFP-TUB1::URA3* (pAFs125)	This study
YSK2874	W303a *bar1* Δ*bfa1::KAN* pRS304*-mCherry-TUB1 (*pAK010-1*)*	Laboratory stock
YSK3448	W303a Δ*bfa1::HIS3* Δ*bub2::URA3 MOB1-GFP::KAN* pRS304*-mCherry-TUB1* (pAK010-1)	This study

**Table 2:** Plasmids used in this study.

Plasmid	Source
pMW(L)20- *pGAL*	Laboratory stock
pMW(L)20- *pGAL -BFA1*	Laboratory stock
pMW(L)20- *pGAL* -*BFA1-D16*	This study
pMW(L)20- *pGAL* -*BFA1*-*D8*	This study
*GFP-TUB1::URA3* (pAFs125)	Beth Lalonde
pMW(L)20- *pGAL* -*BUB2*	This study
pMW(U)20-*pGAL*-*GFP*	Laboratory stock
pMW(U)20-*pGAL*-*GFP-BFA1*	This study
pMW(U)20-*pGAL*-*GFP-BFA1-D16*	This study
pMW(U)20-*pGAL*	Laboratory stock
pMW(U)20-*pGAL-KIN4*	This study
pMW(U)20-*pGAL-SNU13*	This study
pMW(U)20-*pGAL-GFP-SNU13*	This study
pRS304-*BFA1-3HA*	This study
pRS304-*BFA1-D16-3HA*	This study
pMW(L)20- *pGAL -BFA1^2A^*	This study
pMW(L)20- *pGAL* -*BFA1-D16^2A^*	This study
pRS306*-mCherry-TUB1*	D. Liakopoulos
pRS304*-mCherry-TUB1*	E. Schiebel

**Table 3:** Primers and enzymes used for plasmid constructions.

Constructs	Primername	Sequence	Restriction Site
pMW(L)20-*pGAL*-*BFA1*-*D8*	KS1782 (Forward)	GCAGAGCTCATGAAGCAAAAATATTCTGAT	*Sac* I
	KS1783 (Reverse)	CCATGGATCCGCTAAAGGGCTAATCTTTTG	*BamH* I
pMW(L)20-*pGAL*-*BFA1-D16*	KS1784 (Forward)	TTGAGCTCATGTCAATTAGGCCTCTCAC	*Sac* I
	KS1785 (Reverse)	CGCGGATCCCTAAGCTTTCCTGTTTGCGGT	*BamH* I
pMW(L)20- *pGAL*-*BUB2*	KS1825 (Forward)	GCAGAGCTCATGACCTCAATTGAAGAT	*Sac* I
	KS1826 (Reverse)	CGCGCATGCTTACGGTATATATATGTC	*Sph* I
pMW(U)20-*pGAL-SNU13*	KS2132 (Forward)	GATGAGCTCGGATGTCTGCCCCAAACCCAAA GGCT	*Sac* I
	KS2133 (Reverse)	GTCGGATCCTTAAATTAATAAAGTTTCAATCTTG	*BamH* I
pMW(U)20-*pGAL-KIN4*	KS1970 (Forward)	GATGAGCTCGGATGGCTTCTGTACCTAAACG	*Sac* I
	KS1971 (Reverse)	GTCGGATCCTCAAACCCTCATGCTCCTTC	*BamH* I
pRS304-*BFA1-3HA*	KS1337 (Forward)	ACTGGTACCCGGAGCAAGAGATAGTCTGAG	*Kpn* I
	KS2140 (Reverse)	CTCACTAGTTCAGCACTGAGCAGCGTAATCT	*Spe* I
pRS304-*BFA1-D16-3HA*	KS1337 (Forward)	ACTGGTACCCGGAGCAAGAGATAGTCTGAG	*Kpn* I
	KS2140 (Reverse)	CTCACTAGTTCAGCACTGAGCAGCGTAATCT	*Spe* I
pMW(L)20-*pGAL*-*BFA1^S150A^(or BFA1-D16 ^S150A^)*	KS1568 (Forward)	CCAAGGCTAAAGCAACCTAGAGCAATGATGGAATTGAAACCAAAA	NA
	KS1569 (Reverse)	TTTTGGTTTCAATTCCATCATTGCTCTAGGTTGCTTTAGCCTTGG	NA
pMW(L)20-*pGAL*-*BFA1^S180A^(or BFA1-D16 ^S180A^)*	KS1570 (Forward)	AATTCTGTAAGATTTAAGAAAGCAATGCCTAACTTAGCTTTAGTT	NA
	KS1571 (Reverse)	AACTAAAGCTAAGTTAGGCATTGCTTTCTTAAATCTTACAGAATT	NA

## Data Availability

All data are included within the main text and the Supplementary Materials.

## Supplementary Materials

The following supporting information can be downloaded at: https://www.mdpi.com/article/10.3390/cells11142179/s1: Figure S1: The summary of Bfa1 functional domains used in this study; Figure S2: Bfa1-D16 overexpression inhibits MEN signalling pathway to induce anaphase arrest; Figure S3: Bfa1 and Bfa1-D16 overexpression promote growth arrest in various MAP deletion mutants; Figure S4: Bfa1 interacts with Bim1 but does not interfere with Bim1 binding to Kar9 or Kip2; Figure S5: The nuclear position in Δ*bfa1* cells.
