# Supplementary material for: The N-Terminal Domain of Bfa1 Coordinates Mitotic Exit Independent of GAP Activity in Saccharomyces cerevisiae
Source: Cells. 2022 Jul 12;11(14):2179. doi: 10.3390/cells11142179 (PMC9316867; doi:10.3390/cells11142179)
Supplement: Supplementary file 1 [file cells-11-02179-s001.zip › cells-1709930-sup.pdf]

cells

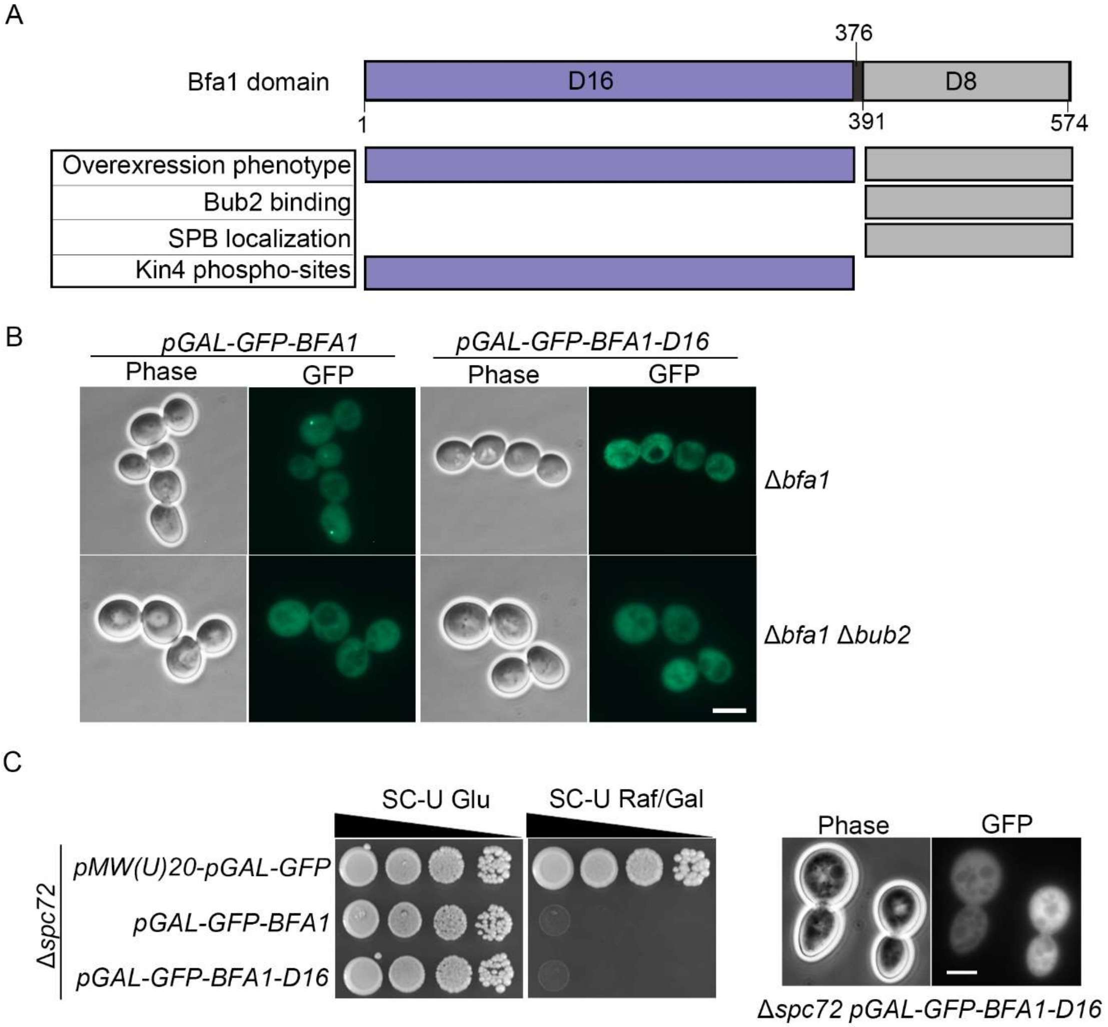

Figure S1. The summary of Bfa1 functional domains used in this study (**A**) The functions of Bfa1-D16 and Bfa1-D8 according to Kim *et al.* (2004). The overexpression phenotype represents the anaphase arrest induced by overexpression of Bfa1-D16 or Bfa1-D8. Bfa1-D16 localizes to the cytoplasm and cannot bind Bub2. The phosphorylation sites (serine 150 and 180) targeted by Kin4 are present in Bfa1-D16. Bfa1-D8 binds Bub2 and localizes to SPBs. (**B**) Localization of GFP-Bfa1 and GFP-Bfa1-D16 in Δ*bfa1* and Δ*bub2* Δ*bfa1* cells, respectively. The Δ*bfa1* (YSK3270) and Δ*bub2* Δ*bfa1* (YSK1879) cells transformed with pMW(U)20-*pGAL-GFP-BFA1* or pMW(U)20-*pGAL-GFP-BFA1-D16* were grown to mid-log phase and incubated with SC-U medium (uracil-depleted) containing 2% galactose to induce the expression of Bfa1 or Bfa1-D16 at 25°C for 3 h. (**C**) The Δ*spc72* (YSK3435) cells transformed with pMW(U)20-*pGAL-GFP*, pMW(U)20-*pGAL-GFP-BFA1,* or pMW(U)20-*pGAL-GFP-BFA1-D16* plasmids were serially diluted (10-fold), spotted on SC-U Glu and SC-U Raf/Gal plates, and incubated at 25°C. GFP-Bfa1-D16 localization was monitored in Δ*spc72* cells (the right panel). The cells were grown to the mid-log phase and incubated in SC-U Raf/Gal at 25°C for 3 h to induce the overexpression of GFP-Bfa1-D16. (**B–C**) Images were captured using an Axioplan2 fluorescence microscope with a 100× objective (Carl Zeiss). Scale bar, 5 μm.

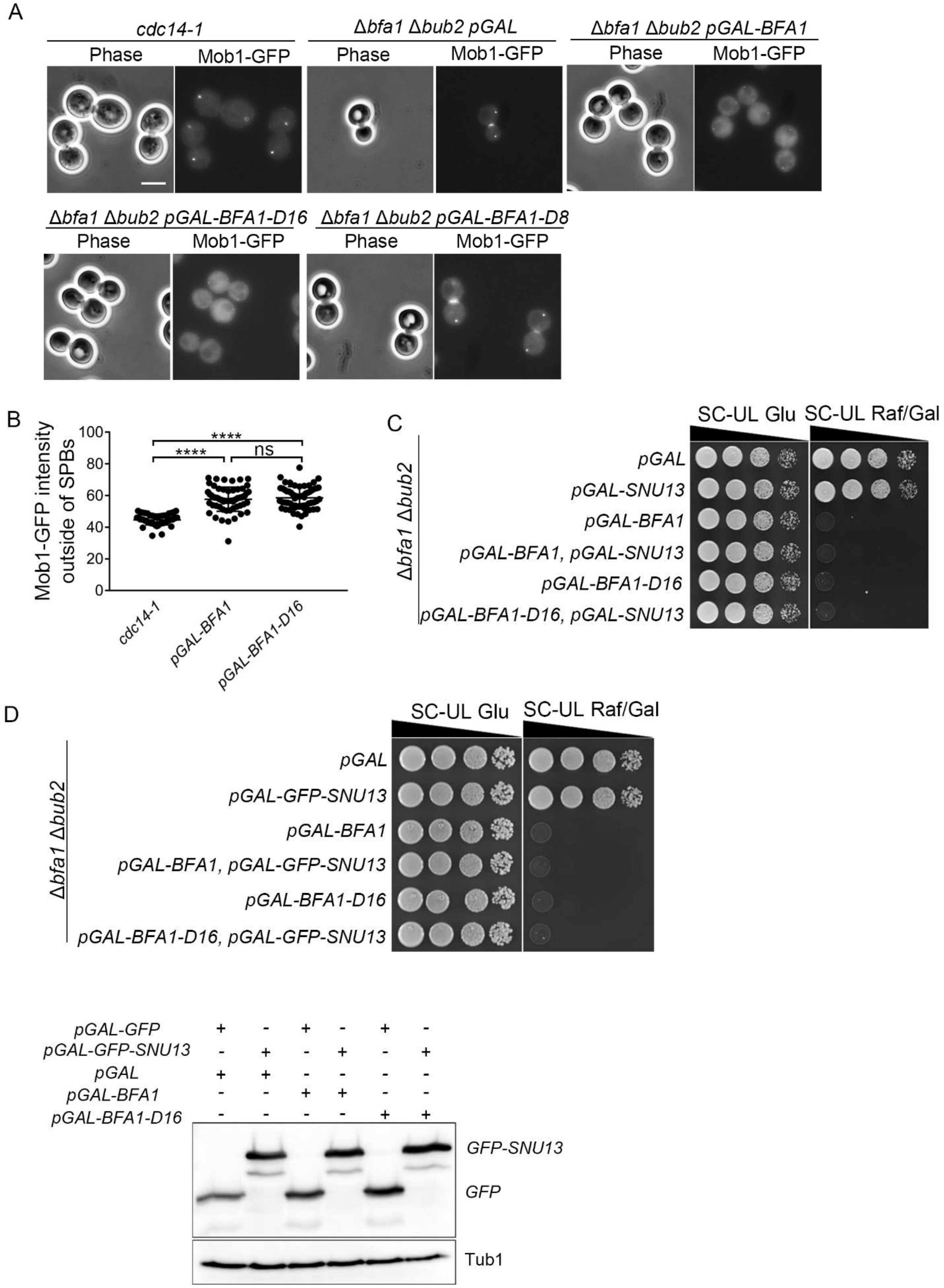
A
cdc14-1
Phase
Mob1-GFP
Δbfa1 Δbub2 pGAL
Phase
Mob1-GFP
Δbfa1 Δbub2 pGAL-BFA1
Phase
Mob1-GFP
Δbfa1 Δbub2 pGAL-BFA1-D16
Phase
Mob1-GFP
Δbfa1 Δbub2 pGAL-BFA1-D8
Phase
Mob1-GFP
B
Mob1-GFP intensity outside of SPBs
100
80
60
40
20
0
****
****
ns
cdc14-1
pGAL-BFA1
pGAL-BFA1-D16
C
SC-UL Glu
SC-UL Raf/Gal
Δbfa1 Δbub2
pGAL
pGAL-SNU13
pGAL-BFA1
pGAL-BFA1, pGAL-SNU13
pGAL-BFA1-D16
pGAL-BFA1-D16, pGAL-SNU13
D
SC-UL Glu
SC-UL Raf/Gal
Δbfa1 Δbub2
pGAL
pGAL-GFP-SNU13
pGAL-BFA1
pGAL-BFA1, pGAL-GFP-SNU13
pGAL-BFA1-D16
pGAL-BFA1-D16, pGAL-GFP-SNU13
pGAL-GFP + - + - + -
pGAL-GFP-SNU13 - + - + - +
pGAL + + - - - -
pGAL-BFA1 - - + + - -
pGAL-BFA1-D16 - - - - + +
GFP-SNU13
GFP
Tub1

Figure S2. Bfa1-D16 overexpression inhibits MEN signaling pathway to induce anaphase arrest. (**A**) Mob1-GFP at SPBs was monitored in Bfa1 or Bfa1-D16-overexpressing Δ*bfa1* Δ*bub2* cells. The Δ*bfa1* Δ*bub2* (YSK3424) strain, with a chromosome containing *GFP*-tagged *MOB1* in its C-terminus, was transformed with pMW(L)20-*pGAL*, pMW(L)20-*pGAL-BFA1,* pMW(L)20-*pGAL-BFA1-D16*, and pMW(L)20-*pGAL-BFA1-D8* plasmids. The indicated cells were incubated with SC-L Raf/Gal at 25°C for 3 h. Mob1-GFP localization of *cdc14-1* cells was used as a positive control of its localization to SPBs. *cdc14-1* (YSK1610) cells harboring *GFP*-tagged *MOB1* in its C-terminus were grown at 25°C and then transferred to 37°C for 3 h. Images were captured using an Axioplan2 fluorescence microscope with a 100× objective (Carl Zeiss). Scale bar, 5 μm. (**B**) Quantification of Mob1-GFP intensity outside of SPBs. The mean intensity of a segmented region (outside of SPBs) with a fixed area (2.37 μm^2) per cell was measured for each genotype (n= 60 for each genotype) by Fiji ImageJ after background correction using BaSiC plugin and plotted. (**C , D**) Overexpression of the snoRNP component Snu13 does not suppress the defective growth mediated by Bfa1 or Bfa1-D16 overexpression. Δ*bfa1* Δ*bub2* cells (YSK1879) transformed with (C) pMW(L)20-*pGAL*, pMW(U)20-*pGAL*, pMW(L)20-*pGAL-BFA1*, pMW(L)20-*pGAL-BFA1-D16,* and/or pMW(U)20-*pGAL-SNU13* and transformed with (D) pMW(L)20-*pGAL*, pMW(U)20-*pGAL-GFP*, pMW(L)20-*pGAL-BFA1*, pMW(L)20-*pGAL-BFA1-D16,* pMW(U)20-*pGAL-GFP*, and/or pMW(U)20-*pGAL-GFP-SNU13* were serially diluted (10-fold), spotted on SC-UL Glu and SC-UL Raf/Gal plates, and incubated at 25°C. (**D**) Identification of GFP-Snu13 expression by a western blot. Tub1 as a loading control.

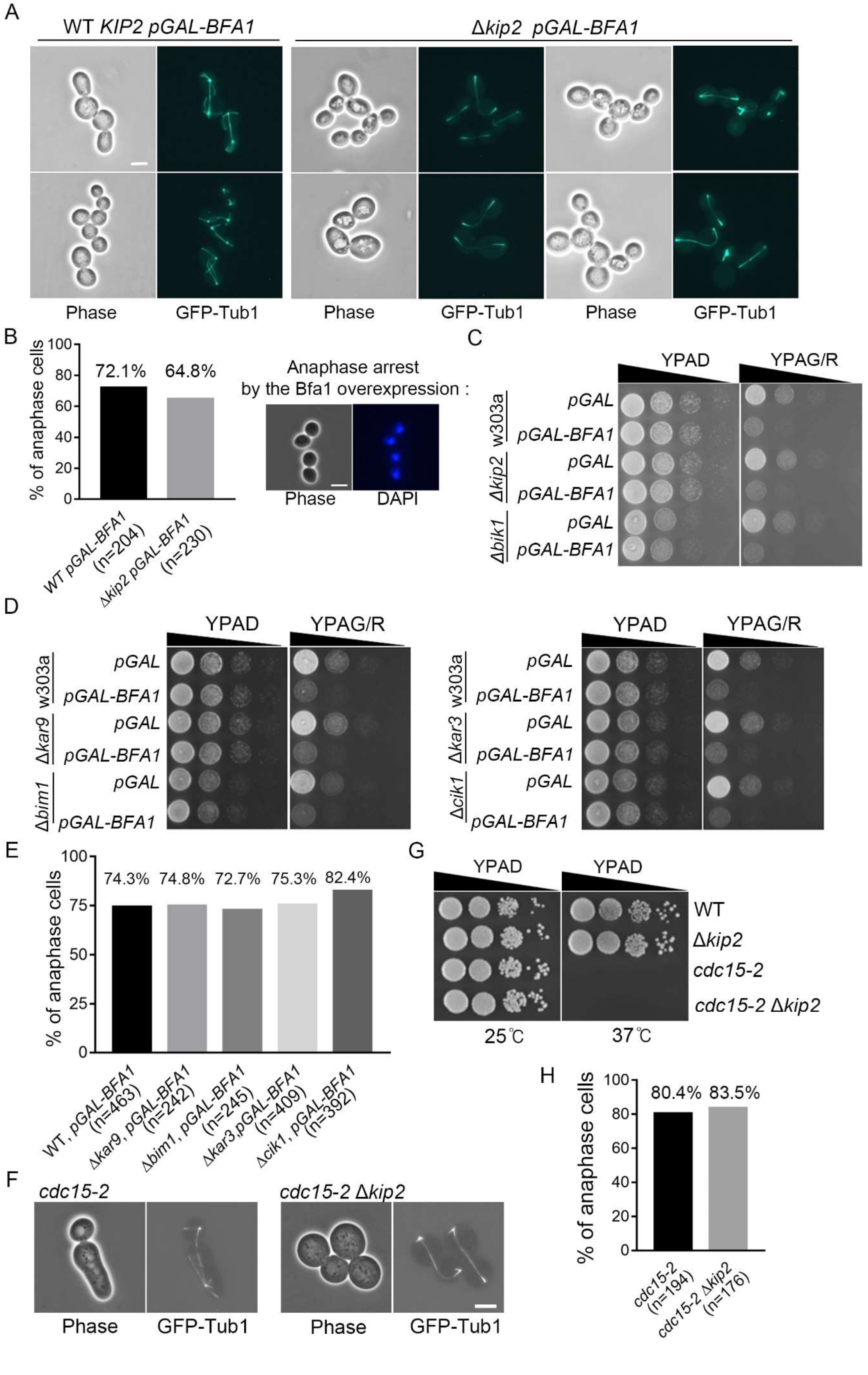
A
WT KIP2 pGAL-BFA1
Δkip2 pGAL-BFA1
Phase
GFP-Tub1
B
% of anaphase cells
72.1%
64.8%
WT pGAL-BFA1 (n=204)
Δkip2 pGAL-BFA1 (n=230)
Anaphase arrest by the Bfa1 overexpression :
Phase
DAPI
C
YPAD
YPAG/R
w303a
Δkip2
Δbik1
pGAL
pGAL-BFA1
D
YPAD
YPAG/R
w303a
Δkar9
Δbim1
Δkar3
Δcik1
pGAL
pGAL-BFA1
E
% of anaphase cells
74.3%
74.8%
72.7%
75.3%
82.4%
WT, pGAL-BFA1 (n=463)
Δkar9, pGAL-BFA1 (n=242)
Δbim1, pGAL-BFA1 (n=245)
Δkar3,pGAL-BFA1 (n=409)
Δcik1, pGAL-BFA1 (n=392)
F
cdc15-2
cdc15-2 Δkip2
Phase
GFP-Tub1
G
YPAD
YPAD
WT
Δkip2
cdc15-2
cdc15-2 Δkip2
25℃
37℃
H
% of anaphase cells
80.4%
83.5%
cdc15-2 (n=194)
cdc15-2 Δkip2 (n=176)

Figure S3. Bfa1 and Bfa1-D16 overexpression promote growth arrest in various MAP deletion mutants. (**A**) Wild-type (WT, YSK3278) and *Δkip2* (YSK3434) strains with chromosomes integrated with *GFP-TUB1* were transformed with pMW(L)20-*pGAL-BFA1* and grown to the mid-log phase. Bfa1 overexpression was induced by incubating the cells in SC-L Raf/Gal for 3 h. GFP-Tub1 images were captured using an Axioplan2 fluorescence microscope with a 100× objective (Carl Zeiss). Scale bar, 5 μm. (**B**) The cells indicated in (A) were stained with DAPI after fixation and sonication. Images of DAPI-stained cells were captured using an Axioplan2 fluorescence microscope with a 100× objective (Carl Zeiss). Scale bar, 5 μm. The percentage of anaphase cells (%) was calculated as the number of anaphase cells relative to the total cells (*n* = 204 and *n* = 230 for each genotype). (**C**) Δ*kip2* (YSK3348) and Δ*bik1* (YSK3349) cells transformed with pMW(L)20-*pGAL* or pMW(U)20-*pGAL-BFA1* in log phase were serially diluted (10-fold), spotted on YPAD and YPAG/R (2% galactose and 2% raffinose) plates, and incubated at 25°C. (**D**) Growth arrest by Bfa1 overexpression was observed in deletion mutants of several MAPs. Wild-type (YSK368), Δ*kar9* (YSK3344), Δ*bim1* (YSK3345), Δ*kar3* (YSK3346), and Δ*cik1* (YSK3347) strains transformed with pMW(L)20-*pGAL* and pMW(U)20-*pGAL-BFA1* were serially diluted (10-fold), spotted on YPAD and YPAG/R plates, and incubated at 25°C. (**E**) The percentage of anaphase arrest cells in (D). Cells in (D) were stained with DAPI after fixation and sonication, and the percentage of anaphase cells was calculated as the number of anaphase cells relative to the total number of cells (*n* = 463, = 242, = 245, = 409, and = 392 for each genotype). (**F**) The MT phenotypes in cells were visualized with GFP-Tub1. The *GFP-TUB1* was integrated into the chromosomes of *cdc15-2* (YSK553) and *cdc15-2* Δ*kip2* (YSK3322) strains. Cells were grown to the mid-log phase at 25°C and transferred to 37°C for 3 h. Images were captured using an Axioplan2 fluorescence microscope with a 100× objective (Carl Zeiss). Scale bar, 5 μm. (**G**) Cells of indicated genotypes (YSK3278, YSK3434, YSK553, YSK3322) grown to the mid-log phase were serially diluted (10-fold), spotted on YPAD plates, and incubated at 25°C and 37°C. (**H**) The cells shown in (F) were stained with DAPI after fixation and sonication. The percentage of anaphase cells (%) was calculated as the number of anaphase cells relative to the total number of cells (*n* = 194 and *n* = 176 for each genotype).

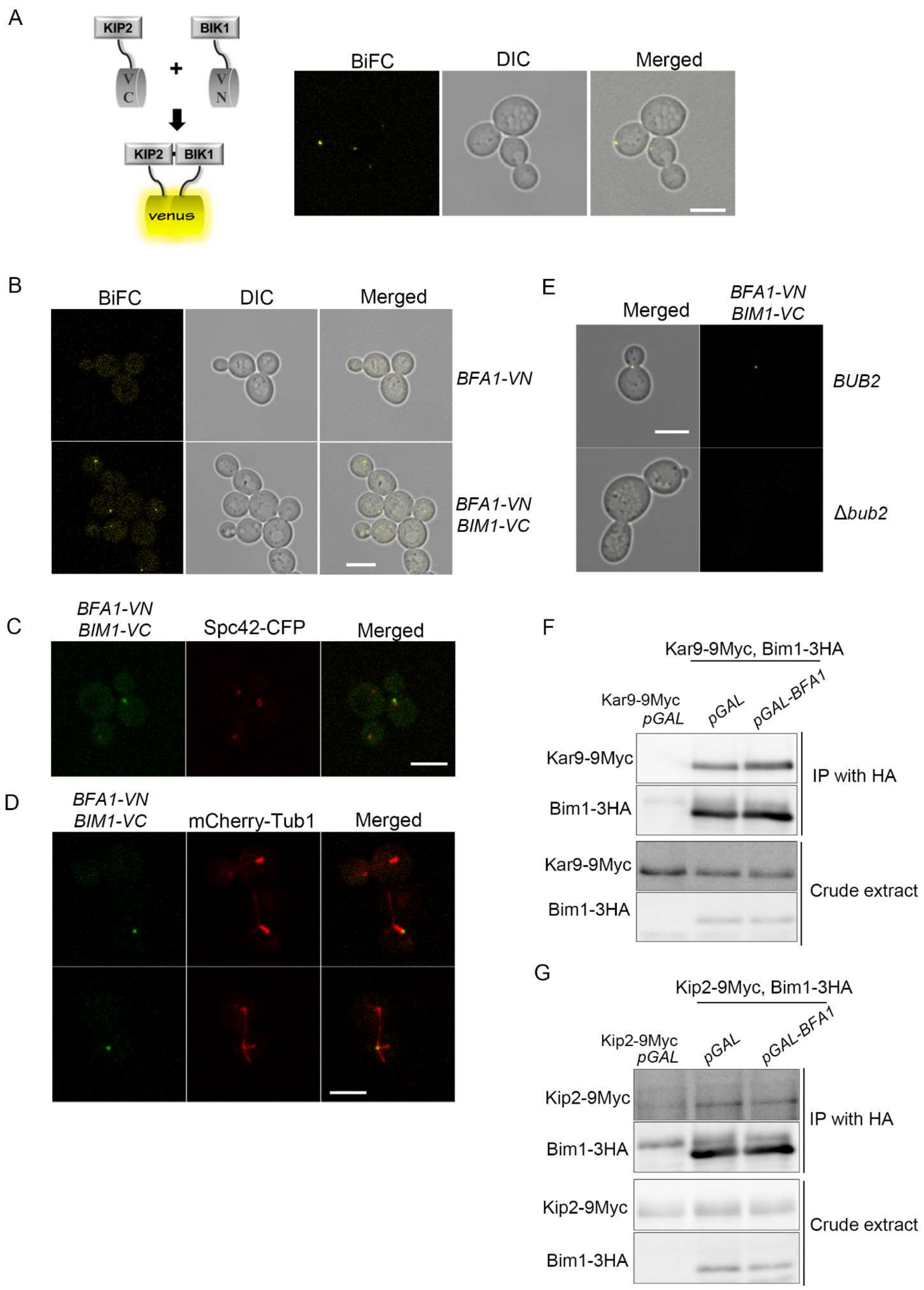

Figure S4. Bfa1 interacts with Bim1 but does not interfere with Bim1 binding to Kar9 or Kip2. (**A**) Bik1–Kip2 interaction according to the BiFC assay. *KIP2* and *BIK1* were respectively tagged in the C-terminus with the C-terminal end of

venus (VC) and the N-terminal end of venus (VN) in the wild-type (YSK3284) chromosome. Two non-fluorescent fragments (VN, VC) were fused to create a yellow fluorescent protein (venus) upon Kip2–Bik1 interaction. The BiFC signals were observed in 79 out of 83 cells. (**B**) The Bfa1–Bim1 interaction according to the BiFC assay. The C-terminus of *BFA1* was tagged with the N-terminal end of venus (VN), and the C-terminus of *BIM1* was tagged with the C-terminal end of venus (VC) (YSK3304). The BiFC interaction signals were observed in 113 of 179 cells through at least three independent experiments. Cells expressing Bfa1-VN alone (YSK3150) were used as negative controls. No signal was detected when 41 cells expressing Bfa1-VN alone. (**C**) Bfa1–Bim1 interaction was observed with the SPB component Spc42. *SPC42* in cells co-expressing Bfa1-VN and Bim1-VC was tagged using *CFP-KAN* at its C-terminus (YSK3359). A total of 17 cells were observed, of which 14 cells showed a BiFC signal, and the signal was colocalized with one of two Spc42-CFP signals indicating SPBs. (**D**) Bfa1–Bim1 interaction was observed according to the BiFC assay in the presence of mCherry-Tub1. Chromosomal DNA of cells co-expressing Bfa1-VN and Bim1-VC was integrated with pRS306-*mCherry-TUB1* (YSK3360). A total of 15 cells were observed, of which 7 cells showed a BiFC signal, and the signal was located at one end of the spindle. (**E**) Bfa1–Bim1 interaction was observed by the BiFC assay in Δ*bub2* cells. The *BUB2* gene in cells expressing Bfa1-VN and Bim1-VC was deleted and replaced with *KanMx6* (YSK3361). No signal was detected when the 28 cells of Δ*bub2* were observed, when the BiFC signal was observed in 22 out of 33 cells with the presence of Bub2. (**A-E**) Images were captured with an LSM 880 confocal microscope with a 100× objective (Carl Zeiss). Scale bar, 5 μm. (**F, G**) Co-IP of Bim1–Kar9 and Bim1–Kip2 interactions in Bfa1-overexpressing cells. Δ*bfa1* cells expressing Kar9-9Myc (YSK3268), Kar9-9Myc and Bim1-3HA (YSK 3269), Kip2-9Myc (YSK3243), and Kip2-9Myc and Bim1-3HA (YSK3244) were transformed with pMW(L)20-*pGAL* or pMW(L)20-*pGAL-BFA1*, respectively, grown to mid-log phase, incubated with SC-L (leucine-depleted) medium containing 2% galactose to induce Bfa1 overexpression, and then lysed for Co-IP. Bim1-3HA was precipitated with anti-HA and Protein A agarose. Kar9-9Myc, Kip2-9Myc, and Bim1-3HA were blotted with anti-Myc and anti-HA antibodies.

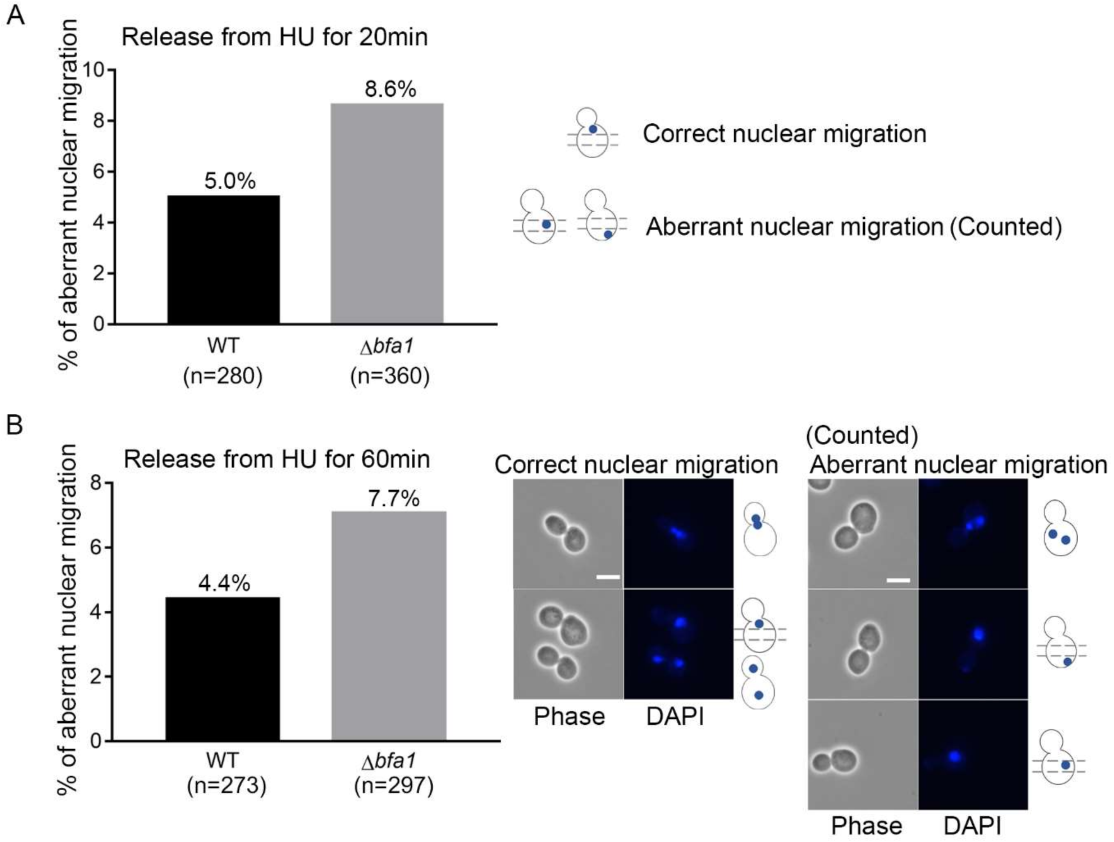

Figure S5. The nuclear position in Δ*bfa1* cells. **(A, B**) Wild-type (WT, YSK3278) and Δ*bfa1* (YSK3279) cells in the log phase were treated with 200 mM hydroxyurea (HU) for 2 h and transferred to YPAD plates for incubation at 30°C for 20 min and 60 min, respectively. Cells were stained with DAPI after fixation and sonication, and the percentage of aberrant nuclear migration was calculated as the number of cells with the indicated phenotypes shown in the figure relative to the total number of cells (*n* = 280, = 360, = 273, and = 297 for each strain, respectively). Images were taken using an Axioplan2 fluorescence microscope with a 100× objective (Carl Zeiss). Scale bar, 5 μm.
